# Cost-Utility Analysis of STN1013001, a Latanoprost Cationic Emulsion, versus Other Latanoprost Formulations (Latanoprost) in Open-Angle Glaucoma or Ocular Hypertension and Ocular Surface Disease in France

**DOI:** 10.1155/2022/3837471

**Published:** 2022-04-29

**Authors:** Carlo Lazzaro, Cécile van Steen, Florent Aptel, Cédric Schweitzer, Luigi Angelillo

**Affiliations:** ^1^Studio di Economia Sanitaria, Milan, Italy; ^2^Santen GmbH, München, Germany; ^3^Department of Ophthalmology, University Hospital, CHU Grenoble-Alpes, Grenoble, France; ^4^CHU Bordeaux, Department of Ophthalmology, University of Bordeaux, 33000 Bordeaux, France; ^5^Inserm, Bordeaux Population Health Research Center, Team LEHA, UMR 1219, 33000 Bordeaux, France

## Abstract

**Purpose:**

To investigate the cost utility of STN1013001, a latanoprost cationic emulsion, versus Latanoprost in patients with open-angle glaucoma or ocular hypertension (OAG/OHT) and concomitant ocular surface disease (OSD) in France.

**Methods:**

An early Markov model, including 7 health states and a 1-year cycle length, was developed to estimate the cost utility of STN1013001 versus Latanoprost from the French health system perspective over a 5-year time horizon. The model was populated with pooled data (treatment adherence, quality of life, disease progression, and resource utilization) collected, via a questionnaire, from a convenience sample of 5 French glaucoma specialists. Remaining data were retrieved from published sources. Half-cycle correction and 2.5% real social discount rate were applied to costs (in €2020), life years saved (LYS), and quality-adjusted life years (QALYs). The incremental cost-utility ratio (ICUR) was contrasted against the informal willingness-to-pay (WTP) range for incremental LYS or QALY gained (€30,000–€50,000) suggested for France. One-way and probabilistic sensitivity analyses tested the robustness of the baseline ICUR.

**Results:**

Over a 5-year time horizon, STN1013001 resulted in an incremental 0.35 QALYs gained at an incremental cost of €7.39 compared to Latanoprost, resulting in an ICUR of €21.26. This is well below the lower limit of the unofficial WTP range proposed for France. Sensitivity analyses confirmed the robustness of the baseline results.

**Conclusion:**

Once on the market, STN1013001 will provide the French health system with a cost-effective treatment versus Latanoprost for OAG/OHT + OSD patients. These results should be confirmed by future economic evaluations carried out alongside empirical trials.

## 1. Introduction

Glaucoma is one of the most frequent causes of irreversible blindness, hence posing a substantial public health problem [1]. The clinical and economic burden of glaucoma are expected to become even more alarming in the future, as the number of patients suffering from glaucoma is projected to rise steeply, affecting approximately 112 million people worldwide by 2040 compared with 63.4 million in 2013 [1–3]. Open-angle glaucoma (OAG) is the most prevalent form of glaucoma, accounting for approximately 86% of all glaucoma diagnoses worldwide [1].

In France, about 500,000 patients are diagnosed with open-angle glaucoma (OAG) or ocular hypertension (OHT). Similar numbers of OAG/OHT patients are expected to be undiagnosed [4].

Glaucoma is a progressive and chronic disease and generally develops according to the following stages: ocular hypertension (OHT, stage 0), early glaucoma (stage 1), moderate glaucoma (stage 2), advanced glaucoma (stage 3), severe glaucoma (stage 4), and end-stage glaucoma/blindness (stage 5) [4–7] (Supplementary Material Table S1).

Patients generally only suffer from glaucoma symptoms once the disease has progressed to its advanced stages and the patient is experiencing irreversible vision loss [7]. Hence, early diagnosis and appropriate treatment are vital to maintain vision in OAG/OHT patients [3].

According to the most recent available data, during a 5-year time span, the annual cost per patient affected by OHT or OAG in France varies between Euros (€)7322 (OHT) and €8488 (OAG) (values expressed in €2000–€2004) [8].

Despite existing treatment options, low treatment adherence rates remain an important barrier to effective OAG/OHT treatment [9, 10].

In addition, a significant part of OAG/OHT patients (35%–65%, depending on the number of hypotensive drops administered) experience ocular surface disease (OSD) and related symptoms [11]. The effective management of OSD symptoms, including dry eye disease (DED), remains an unmet therapeutic need [12, 13].

The concomitant OSD symptoms can dramatically affect patients' activities of daily living, notably lower their adherence and persistence to OAG/OHT therapies, and reduce their health-related quality of life (HRQoL), also known as utility (that usually ranges between 0, i.e., death or HRQoL perceived worse than death and 1, i.e., perfect health, bounds included) [14–16]. Insufficient effective management of concomitant OSD can deteriorate IOP control and increase the risk of OAG/OHT progression [17]. Hence, there is a strong case for new therapeutic options that address these unmet needs and help avoid irreversible vision loss.

STN1013001 (Santen, Osaka, Japan), formerly DE-130A, is a latanoprost cationic emulsion for the treatment of OAG/OHT with concomitant OSD [18]. The cationic nanoemulsion (Novasorb® technology) possesses tear film stabilization and anti-inflammatory properties, representing a technical innovation for the effective management OAG/OHT with concomitant OSD [13].

In a 3-month Phase 2 trial, STN1013001 proved similar to Latanoprost at reducing IOP (−6.0% vs. −5.4%; *p* > 0.05) and significantly reduced both OSD-related signs and symptoms (−36.0% vs. −7.0%; *p* < 0.05, respectively) versus baseline in the per protocol study population [19].

As a head-to-head Phase III trial of STN1013001 versus Latanoprost is ongoing (NCT04133311) [20], STN1013001 is not yet available on the French market.

In this instance, early-stage health economic models help assess the economic value of drugs that are in development [21, 22].

This paper reports on an early Markov model-supported cost-utility analysis (CUA) [14–16, 23] comparing STN1013001 to other latanoprost formulations that are currently available in France (Latanoprost) in notional OAG/OHT + OSD patients, following the national health system perspective [15, 16].

The aim of this early health economic model is to anticipate evidence about the sustainability of STN1013001 for the French health system before it enters the market [21, 22].

## 2. Material and Methods

### 2.1. Decision Model

**Figure 1:**
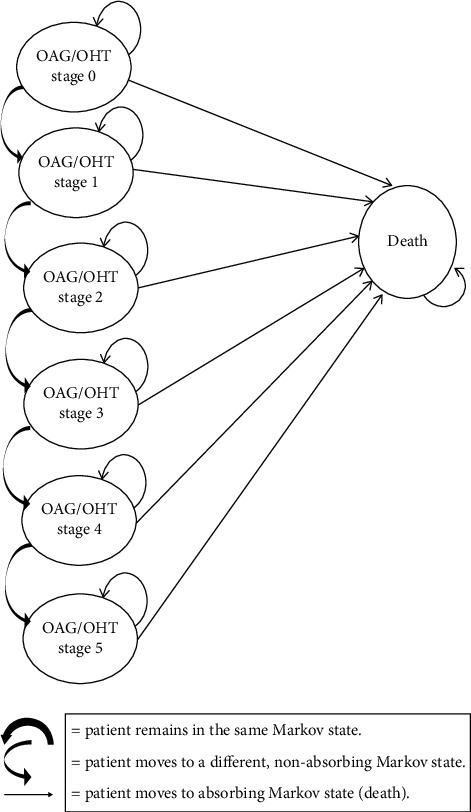
Markov model. OAG/OHT = open-angle glaucoma or ocular hypertension.

The Markov model includes 7 mutually exclusive health states (OAG/OHT stages 0–5 and gender and age-specific all-cause mortality) [4, 6, 23, 24]. It compares two hypothetical cohorts of patients on STN1013001 or Latanoprost (1000 notional patients each) over a 5-year time horizon, adopting a 1-year cycle length (365.25 days per cycle to take leap years into account) [25] (Figure 1).

The Markov model, which consisted of 904 parameters, was mainly populated with pooled data obtained from a convenience sample [26] of 5 French ophthalmologists with extensive experience in diagnosing and treating OAG/OHT (on average 902 patients followed up per year). The ophthalmologists practice in various settings (teaching hospital: 3; private office: 1; and private eye clinic: 1). Remaining data were obtained from the literature.

For both the hypothetical cohorts, OAG/OHT stage 0 was the starting Markov health state. At each Markov cycle, notional patients can remain in the same OAG/OHT stage, move to more severe OAG/OHT stages, or pass away (all cause-mortality) according to a transition probability matrix [14–16, 23] (Table S2). In line with the expected progression of the disease, backward transitions from more to less severe OAG/OHT stages were not allowed.

Costs, life years saved (LYS) and quality-adjusted life years (QALYs) were discounted at 2.50% real social discount rate [14–16, 27]. The real social discount rate was set at 0% and 4.5% in one-way sensitivity analysis (OWSA) [14–16, 27].

The half-cycle correction (i.e., the assumption that notional patients accrue 6-month costs, LYS and QALYs the year they die) was applied [14, 23].

The Markov model-supported CUA was created with Excel per Windows® 2010 (Microsoft, Redmond, WA, USA).

#### 2.1.1. Data Collection

Between July and December 2020, a questionnaire and the target product profile of STN1013001 were sent out by e-mail to the 5 French glaucoma experts from high-volume care practice centres.

The glaucoma specialists were requested to complete the questionnaire based on the target product profile of STN1013001 as well as their previous experience with Latanoprost.

The following OAG/OHT stage-specific data were collected for both hypothetical cohorts of patients according to ophthalmologists' opinion [28]: expected number of patients on STN1013001 or Latanoprost per year, annual probabilities of remaining in the same OAG/OHT stage or moving to a more severe one, annual treatment adherence probabilities, HRQoL (utility values), and healthcare resource utilization associated with the diagnosis and management and follow-up of OAG/OHT and OSD.

All questionnaires were returned complete. When necessary, follow-up teleconferences were scheduled with the ophthalmologists for clarifications or missing data management.

The annual probability of OSD (STN1013001 = 0.762; Latanoprost = 0.837; *p* > 0.05) was obtained from the Phase II trial comparing STN1013001 versus Latanoprost [19].

#### 2.1.2. Quality-Adjusted Life Years

QALYs were obtained by multiplying LYS by OAG/OHT stage-specific utility and OSD-related disutility values. The latter was assumed to be the same for notional patients on STN1013001 or Latanoprost and was assumed to be equal to the disutility associated with severe DED (−0.120), in line with clinical practice [29]. Utility values were either obtained from literature (OAG/OHT stages 0 and 5) [30] and were assumed to be the same for both the hypothetical cohorts of patients. Utility values for OAG/OHT stages 1–4 were elicited from the experts, who were assumed to be good proxies given their extensive experience [28] (Table 1).

Utility value for death was set at 0 [15, 16].

### 2.2. Cost of STN1013001 and Latanoprost

In line with the French health system perspective adopted in this CUA, the following cost items were included in the Markov model: STN1013001, Latanoprost, add-on therapies in case of insufficient IOP control on STN1013001 or Latanoprost monotherapy, drugs for OSD management; tests and specialist visits for OAG/OHT diagnosis, patients' follow-up, and OSD management.

The treatment duration of one pack of STN1013001 was assumed to cover, on average, 30 days of treatment. One pack of Latanoprost was assumed to last 29.73 days, taking into account the availability of latanoprost formulations covering 30 days (30 unit dose containers) and 28 days (multidose bottles which are generally discarded 28 days after opening).

Following a similar approach, a 29-day and a 30-day mean treatment duration were estimated for add-on therapies and OSD-related drugs, respectively.

STN1013001 and Latanoprost therapies were assumed to last 365.25 days a year.

The unit cost per diem for STN1013001 was calculated on the grounds of its estimated ex-factory price provided by Santen (Table 1). The unit cost per diem for Latanoprost was based on the ex-factory price per month of treatment of all latanoprost formulations currently available on the French market in November 2020 [32], weighted for their current market share [31]. The market shares were calculated based on IQVIA MIDAS Sales data to moving annual total 3rd quarter of 2020. The same approach was adopted to calculate the unit costs per diem of OSD-related drugs and add-on therapies, such as timolol, in case of insufficient IOP control on STN1013001 or Latanoprost monotherapy.

Since all drugs are self-administered by the patient at home, drug administrations were not costed.

Tests and specialist visits for OAG/OHT diagnosis, patients' follow-up, and OSD management were valued at the current outpatient tariffs [33], which were assumed to be acceptable proxies for the real costs borne by the healthcare facilities to provide those healthcare services [35].

Cost were expressed in €2020 values.

### 2.3. Cost-Utility Analysis

In CUA, the difference in costs (incremental cost—ΔC) of STN1013001 and Latanoprost is divided by the difference in QALYs (incremental QALYs—ΔQALYs) [15, 16]. The CUA results are summarized in the incremental cost-utility ratio (ICUR) [15, 16].

The ICUR informs the third-party payer about the sustainability for the healthcare budget of the cost per QALY of a given healthcare programme versus comparator.

This study was conducted in line with the tenets of the Declaration of Helsinki [36].

Since no patient was enrolled in the present CUA [14, 34], no Ethics Committee approval of the study protocol (questionnaire included) was needed according to the current French legislation [37].

### 2.4. Statistical Analysis

The mean number (standard deviation—SD) of notional patients in each Markov health state was calculated.

Most of the parameters included in the Markov model were assigned a statistical distribution [14, 34].

The beta distribution was fitted to dichotomous events (i.e., OAG/OHT patient gender) and OAG/OHT stage-specific utility values, whereas the Dirichlet distribution was assigned to polytomous events (i.e., transition probabilities from less severe to more severe stages of OAG/OHT).

The gamma distribution was fitted to the volume of healthcare resources other than drugs as well as to OSD-related disutility value.

Finally, the normal distribution was fitted to unit cost of healthcare resources (if different from drugs).

The standard error of the mean was computed from the data collected or by imposing a coefficient of variation [38] on the sample estimates [14, 34].

The 95% confidence interval (95% CI) was calculated via the percentile method [34] for parameters that were assigned a theoretical probability distribution as well as for cost, LYS, QALYs, and a set of probabilities (treatment adherence, tests and specialist visits related to the diagnosis and management and follow-up of OAG/OHT and OSD, as well as OSD-related medications).

For parameters that were not given a statistical distribution, a range accompanied the mean.

### 2.5. Sensitivity Analyses

OWSA and probabilistic sensitivity analyses (PSA) were run to check the robustness of the baseline ICUR [14–16, 34].

#### 2.5.1. One-Way Sensitivity Analysis

OWSA was performed on one parameter at a time by replacing its sample estimate with the lower and upper limits of its 95% CI or range [15, 16]. OWSA results were depicted on a Tornado diagram [15].

#### 2.5.2. Probabilistic Sensitivity Analysis

PSA [14–16, 34, 39] investigated the uncertainty of the baseline ICUR via a 10,000-iteration Monte Carlo simulation [14–16, 34].

During each Monte Carlo iteration, a random value for each probability parameter was sampled [14, 34].

For methodological reasons, the reference literature on health economic modelling [34] recommends not to include parameters that are set by clinical guidelines and national regulatory agencies in the PSA, as these parameters are not subject to uncertainty. Consequently, the following parameters were not included in the PSA: drug posology and cost; real social discount rate for costs, LYS and QALYs (in line with the French guidelines on the economic evaluation of healthcare programmes) [27].

The conjoint density of ΔC and ΔQALYs provided by the MC simulation were reported on the cost-effectiveness plane (CEP) (SText definition S1) [40].

Once expressed in the Net Monetary Benefit (NMB) metric (SText definition S2) [14–16, 34, 39, 41, 42], PSA results were reported graphically via the nonparametric cost-effectiveness acceptability curve (CEAC) (SText definition S3) and cost-effectiveness acceptability frontier (CEAF) (SText definition S4). Given a set of threshold values decided by the third-party payer, the CEAC provides a graphical representation of the probability that a given healthcare programme is cost-effective, whereas the CEAF indicates which health technology is optimal given its expected highest NMB [14–16, 34, 43, 44].

## 3. Results

### 3.1. Markov Model

Both the hypothetical cohorts of patients were assumed to enter the Markov model in OAG/OHT—stage 0 at the age of 47.31 years (range: 45.00; 55.00 years) (Table S3).

**Figure 2:**
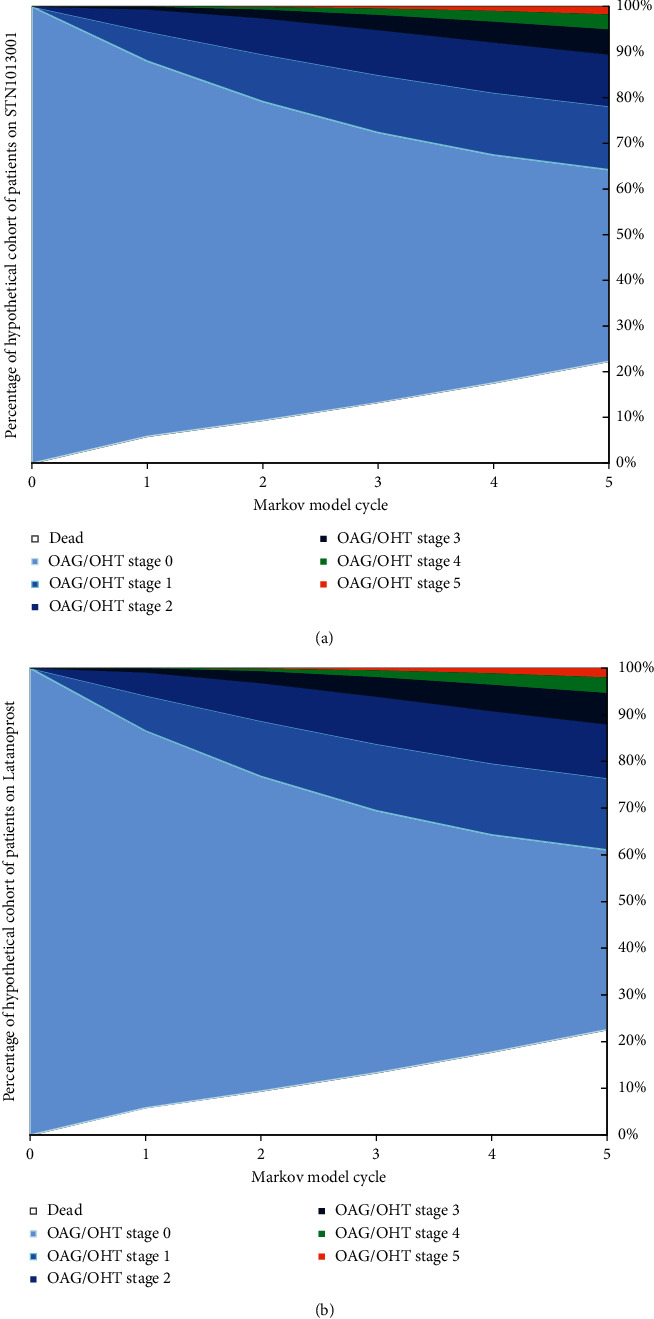
(a) Base case analysis–results–Markov trace–hypothetical cohort of patients on STN1013001. (b). Base case analysis–results–Markov trace–hypothetical cohort of patients on Latanoprost. OAG/OHT = open-angle glaucoma or ocular hypertension.

During the 5-year time horizon, approximately 65% of notional patients on STN1013001 or Latanoprost remained in OAG/OHT stage 0 (STN1013001: mean: 672; SD: 215; Latanoprost: mean: 649; SD: 228) (Figures 2(a) and 2(b); Table S4). A small fraction of both the hypothetical cohorts of patients eventually moved to OAG/OHT stage 5 (STN1013001: mean: 5; SD: 7; Latanoprost: mean: 6; SD: 8) All-cause mortality was similar in both hypothetical cohorts (STN1013001: mean: 114 deaths; SD: 81; Latanoprost: mean: 115 deaths; SD: 82).

### 3.2. Adherence to STN1013001 and Latanoprost

Notional patients on STN1013001 reported higher treatment adherence probabilities compared to their Latanoprost counterparts across OAG/OHT stages 0–3 (Table S5). The probability of being treatment adherent is 0.080 (95% CI: 0.048; 0.113) higher in OAG/OHT stage 0 in year 2 and 0.088 (95% CI: 0.055; 0.122) higher in year 4 in STN1013001 notional patients compared to Latanoprost notional patients. In OAG/OHT stage 1 notional patients on STN1013001 report a 0.066 (95% CI: 0.027; 0.105) and 0.098 (95% CI: 0.060; 0.135) higher treatment adherence probability versus Latanoprost in years 5 and 4, respectively. A similar result was observed in OAG/OHT stage 3: adherence probabilities in patients in the STN1013001 hypothetical cohort were 0.047 (95% CI: 0.010; 0.083) and 0.081 (95% CI: 0.040; 0.123) higher for years 1 and 5, respectively, versus their Latanoprost counterparts.

For OAG/OG stages 4 and 5, the adherence probabilities were similar for both medications.

### 3.3. Base Case Analysis

#### 3.3.1. Healthcare Resources Consumption

*Diagnosis*. Healthcare resource utilization associated with the diagnosis of OAG/OHT was similar in both the STN1013001 and Latanoprost hypothetical cohorts (Table S6).

Slit lamp examination and tonometry were the most frequently performed tests for the diagnosis of OAG/OHT (STN1013001 : 100.00%; mean: 1.18; 95% CI: 0.83; 1.59; Latanoprost: 100.00%; mean: 1.19; 95% CI: 0.97; 1.43 for both tests).

All notional patients undergo at least one ophthalmologist visit (STN1013001: mean: 1.24; 95% CI: 1.02; 1.49; Latanoprost: mean: 1.26; 95% CI: 1.05; 1.49).

#### 3.3.2. Add-On Therapies and Follow-Up

According to experts' opinion, STN1013001 and Latanoprost notional patients have the same annual probability of requiring ≥1 add-on therapies due to insufficient control of IOP on STN1013001 or Latanoprost monotherapy. This probability ranges from 40% (OAG/OHT stage 0) to 95% (OAG/OHT 5) (Table S7). Timolol (35% in OAG/OHT stage 0; 50% in OAG/OHT stages 1 and 3; 60% in OAG/OHT stages 2 and 4) and dorzolamide (15% in OAG/OHT stages 3 and 4; 20% in OAG/OHT stages 1 and 2; 45% in OAG/OHT stage 0) were the most frequently prescribed add-on therapies.

Healthcare resource utilization associated with the management and follow-up of OAG/OHT patients was similar for both STN1013001 and Latanoprost patients (Table S8).

Based on expert opinion, all patients in both hypothetical cohorts (stages 0–5) underwent at least 2 follow-up visits with an ophthalmologist. During the follow-up visit, a slit lamp examination and tonometry were performed in all patients.

#### 3.3.3. Management of OSD

Across all disease stages, notional patients in the STN1013001 cohort were less likely to require medication for the management of OSD versus Latanoprost notional patients (Table S9). A statistically significantly lower proportion of notional patients in the STN1013001 cohort required preservative-free lubricants compared to the Latanoprost cohort, across all disease stages. Differences in prescription probabilities for preservative-free lubricants ranged from −5.88% (95% CI: −9.13%; −0.03%) to −11.34% (95% CI: −17.68%; −0.05%) in OAG/OHT stages 0 and 5, respectively. Additionally, ciclosporin was less likely to be required for OSD management in notional STN1013001 patients versus notional Latanoprost patients. Differences ranged from −4.31% (95% CI: −7.46%; −0.01%) to −5.39% (95% CI: −10.46%; −0.003%) in OAG/OHT stages 4 and 5, respectively.

Across all the disease stages, notional patients on STN1013001 were projected to have a lower probability of receiving a Schirmer test versus those on Latanoprost, ranging from −9.57% (95% CI: −15.93%; −3.11%) in OAG/OHT stage 5 to −7.29% (95% CI: −10.35%; −4.23%) in OAG/OHT stage 2, respectively (Table S10).

All notional patients across all disease stages received at least one slit lamp examination and two ophthalmologist visits for the management of OSD.

#### 3.3.4. Cost of STN1013001 and Latanoprost

After 5 years, the mean costs per notional patient on STN1013001 or Latanoprost were similar (€2347.74 vs. €2340.34; ΔC: €7.39; 95% CI: −€159.39; €166.60) (Table 2).

The cost drivers were the healthcare resources consumed due to add-on therapies and follow-up (STN101300: 56.97%; Latanoprost: 57.23%) and OSD management (STN101300: 20.33%; Latanoprost: 24.20%).

Higher cost for OAG/OHT monotherapy totaled by notional patients on STN1013001 versus notional Latanoprost patients (€341.28 vs. €241.81; difference: €99.47; 95% CI: €95.04; €104.53) is partially offset by lower costs in terms of OSD management (€477.27 vs. €566.38; difference: −€89.11; 95% CI: −€160.16; −€19.73).

Additionally, the mean 5-year cost for notional patients on STN1013001 is lower versus Latanoprost in OAG/OHT stages 4 (STN1013001: €2712.87; Latanoprost: €2912.55; difference: −€199.68; 95% CI: −€361.20; −€38.71) and 5 (STN1013001: €2895.87; Latanoprost: €3144.02; difference: −€248.16; 95% CI: −€471.90; −€30.39) (Figure S1).

#### 3.3.5. Cost-Utility Analysis

The ICUR of €21.26 per incremental QALY gained, resulting from an incremental QALY gain of 0.348 at an incremental cost of €7.39 for STN1013001, is well below the lower limit of the unofficial WTP range (€30,000–€50,000) proposed for France [45] (Table 2).

Notional patients on STN1013001 were estimated to gain statistically significantly more QALYs versus Latanoprost over the 5-year time horizon (2.539 vs. 2.191; ΔQALYs: 0.348; 95% CI: 0.285; 0.408) (Table 2).

Across all disease stages, higher QALYs were observed in the STN1013001 hypothetical cohort vs. the Latanoprost hypothetical cohort. This was most apparent in OAG/OHT stage 1 (ΔQALYs: 0.803; 95% CI: 0.716; 0.892) (Figure S2).

Over the 5-year time horizon, both hypothetical cohorts of patients totaled similar average LYS (STN1013001: 4.228; Latanoprost: 4.223; and ΔLYS: 0.005; 95% CI: −0.013; 0.023).

### 3.4. Sensitivity Analyses

#### 3.4.1. One-Way Sensitivity Analysis

**Figure 3:**
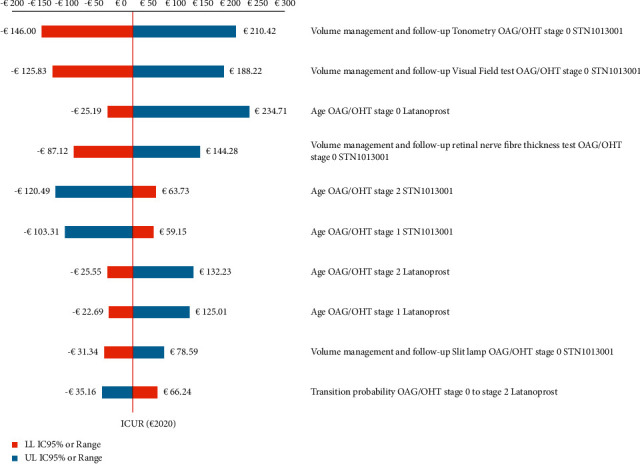
One-way sensitivity analysis–results concerning the first 10 parameters of the Markov model that causes the widest variations in base case ICUR STN1013001 (€21.26; NE sector of the cost-effectiveness plane) (€2020)^a^. ^a^*Y*- and *X*-axes intersect at the baseline ICUR. ICUR = incremental cost-utility ratio; LL 95% CI = lower limit 95% confidence interval; NE = north-east; OAG/OHT = open-angle glaucoma or ocular hypertension; and UL 95% CI = upper limit 95% confidence interval.

OWSA results confirm the robustness of the base case findings. For all the hypotheses tested, STN1013001 remains highly cost-effective or strongly dominant (i.e., being less costly and producing more QALYs) versus Latanoprost, as the highest ICUR does not exceed €235 per QALY gained (Figure 3).

OWSA proves the baseline ICUR to be most sensitive to changes in the volume of follow-up tonometry (−786.85% to +889.91% vs. baseline ICUR) and visual field test (from −691.95% to +785.46% vs. baseline ICUR) performed in OAG/OHT stage 0 STN1013001 notional patients. Additionally, OWSA proves the baseline ICUR to be robust to variations in the social discount rate for costs, LYS and QALYs.

#### 3.4.2. Probabilistic Sensitivity Analysis

**Figure 4:**
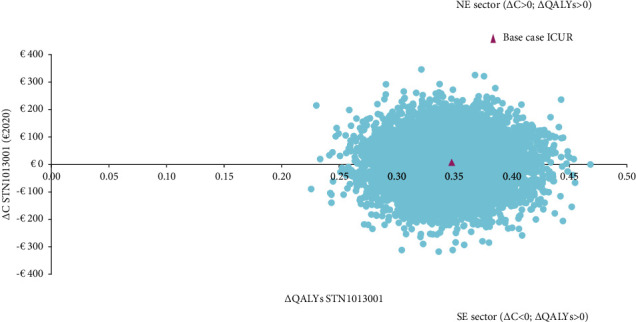
Probabilistic sensitivity analysis-I-cost-effectiveness plane (10,000 out of 10,000 Monte Carlo iterations reported) (€2020). Base case ICUR STN1013001: €21.26 (NE sector of the cost-effectiveness plane). Number of Monte Carlo iterations (%) for each sector of the cost-effectiveness plane: NE = 5088 (50.88%); SE = 4912 (49.12%). ΔC = incremental cost; ΔQALYs = incremental quality-adjusted life years; ICUR = incremental cost-utility ratio; NE = north-east; and SE = south-east.

**Figure 5:**
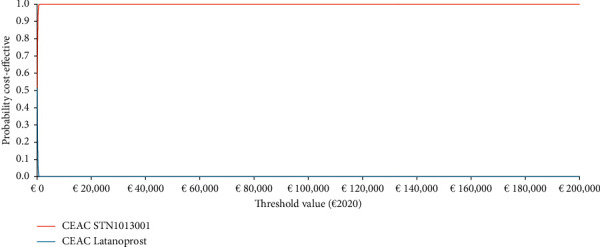
Probabilistic sensitivity analysis-II-cost-effectiveness acceptability curve (1000 out of 1000 threshold values reported) (€2020). Base case ICUR STN1013001: €21.26 (NE sector of the cost-effectiveness plane). CEAC = cost-effectiveness acceptability curve; ICUR = incremental cost-utility ratio; and NE = north-east.

Probabilistic sensitivity analysis shows that STN1013001 has a 49.12% probability of being strongly dominant (i.e., less costly and more effective) versus Latanoprost (Figure 4). The probability that STN1013001 is cost-effective versus the lower bound of the last available unofficial acceptability range for QALY gained (€30,000–€50,000) suggested for the French health system equals 100.00% [45] (Figure 5).

**Figure 6:**
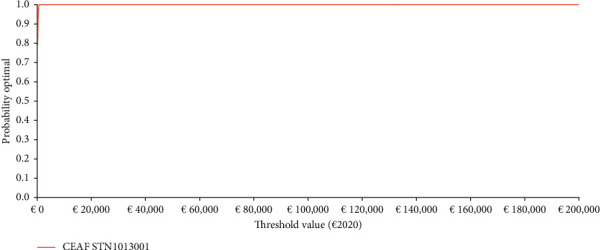
Probabilistic sensitivity analysis-III-cost-effectiveness acceptability frontier (1000 out of 1000 threshold values reported) (€2020). Base case ICUR STN1013001: €21.26 (NE sector of the cost-effectiveness plane). CEAF : STN1013001 is the optimal strategy from a threshold value of €200.20 onwards. CEAF = cost-effectiveness acceptability frontier; ICUR = incremental cost-utility ratio; and NE = north-east.

The CEAF highlights that the probability for STN1013001 to be the optimal alternative (i.e., the healthcare programme with the highest average NMB) for OAG/OHT + OSD patients ranges from 79.01% at a willingness to pay (WTP) for ΔQALY gained = €200.20 to 100.00% from a WTP of €1000 onward (Figure 6).

## 4. Discussion

The present study aimed to obtain a first cost-utility outline of STN1013001 versus Latanoprost for the French setting in OAG/OHT + OSD patients via a Markov model-supported CUA.

Its results demonstrated that, across a 5-year time horizon, STN1013001 is a highly cost-effective therapeutic alternative for this patient population versus Latanoprost from the French health system perspective. This was mainly driven by STN1013001's projected favorable safety profile versus Latanoprost in terms of OSD and consequent improvements in patients' HRQoL and treatment adherence. Despite currently available therapeutic options for the treatment of OAG/OHT, treatment adherence rates remain low and scant evidence is available on the effectiveness of interventions that can improve it [46]. Improving low treatment adherence represents a substantial therapeutic challenge in this patient population. Additionally, the effective management of concomitant OSD is a therapeutic goal that is yet to be met in OAG/OHT patients. This research has shown that STN1013001 can potentially play an important role in addressing both these issues.

Patients in the STN1013001 hypothetical cohort were less likely to develop OSD and, consequently, incurred less costs associated with OSD management (e.g., specialist visits and prescription of preservative-free lubricants) versus Latanoprost notional patients. In line with previous research [7], effectively managing OSD in OAG/OHT patients was expected to significantly reduce the well-known negative impact of OSD on patients' treatment adherence and HRQoL. This was confirmed in the present study, in which higher treatment adherence probabilities were estimated in the STN1013001 hypothetical cohort versus Latanoprost based on experts' opinion. The importance of this is underlined by the fact that high treatment adherence is a reliable predictor of effectiveness and cost-effectiveness of OAG/OHT drugs [47]. The improved treatment adherence, as well as the lower likelihood of developing OSD estimated in the hypothetical cohort of patients on STN1013001, resulted in substantial improvements in HRQoL versus notional patients on Latanoprost.

Another factor that contributed to the higher number of QALYs totaled in the STN1013001 hypothetical cohort was the higher number of patients in less advanced disease stages (stages 0 and 1) compared to the Latanoprost hypothetical cohort across the 5-year time horizon. In accordance with previous research [29], patients in less severe disease stages were assigned a higher utility than notional patients who were partitioned among more severe disease stages, hence contributing to the higher utility values observed in the STN1013001 hypothetical cohort.

Sensitivity analyses confirmed the base case results.

Regardless of the WTP-threshold, the CEP highlights that STN1013001 always produces higher QALYs than Latanoprost.

CEAC and CEAF show the probability for STN1013001 to be cost-effective or optimal at the entire informal acceptability range of €30,000–€50,000 per QALY gained [45] to be 100.00%.

Consequently, the probability of healthcare resources misallocation for the French health system due to STN1013001 funding is zero, even at very low threshold values per QALY gained.

The present research has the following limitations.

First, as STN1013001 is currently not yet available on the French market, an empirical CUA versus Latanoprost was unfeasible. In this scenario, early health economic models are a valuable methodological option for supporting OAG/OHT healthcare decision-making [21, 22, 48–50].

In the literature, two Markov model-based health economic evaluations investigating the cost-effectiveness of Latanoprost in the French setting were identified. One compared Travoprost versus Latanoprost [51], whereas the second compared Travoprost to Latanoprost + Timolol [52]. Similar to the present study, both cost-effectiveness analyses adopted a 5-year time horizon. However, given important differences in the chosen patient population, comparators and outcomes (i.e., IOP reduction was used as an outcome in one of the health economic evaluations [51]), a meaningful comparison with the present study was unfeasible.

A second limitation is related to OAG/OHT stage-specific utility values. The utility values for OAG/OHT stages 0 and 5, that were taken from literature [29], represented the lower and the upper bounds of the scale and might have influenced the remaining OAG/OHT stage-specific utility values provided by experts. However, the adopted approach is similar to the usual layout of the visual analogue scale (VAS), which requests subjects to express their current health state in between the provided bounds of the VAS [15]. Furthermore, given their extensive experience in managing OAG/OHT, the glaucoma specialists were assumed to be reliable proxies for patients' utility. Additionally, comprehensive sensitivity analyses demonstrated that potential uncertainty surrounding the estimated utility values did not impact the base case results.

Lastly, in the present study, surgical or laser treatment was not considered as an alternative to OAG/OHT medications [48]. The rationale supporting this exclusion rests on the fact that laser therapy and surgical procedures are generally performed as second-line treatment options, after topical IOP-lowering eye drops have failed to sufficiently control IOP.

In conclusion, our results demonstrate that STN1013001 will provide the French health system with a cost-effective therapy versus Latanoprost for OAG/OHT + OSD patients.

These findings should be confirmed empirically when STN1013001 is available to patients in France.

## Figures and Tables

**Table 1:** Base case analysis–methods–unit cost for healthcare resources, utility, and disutility values (95% CI)^a,b^ (costs in €2020).

Model main items	Point estimate (95% CI)		Source
**OAG/OHT stages 0-5**			
**Drugs**			
**OAG/OHT medications** ^**c,d**^			
STN1013001	€0.30		Santen GmbH2020
Latanoprost	€0.24		IQVIA [31], Ministère des Affaires Sociales et de la Santé [32]
**Add-on therapies** ^**d,e**^			
Acetazolamide	€0.16		IQVIA [31], Ministère des Affaires Sociales et de la Santé [32]
Brimonidine	€0.12		IQVIA [31], Ministère des Affaires Sociales et de la Santé [32]
Brinzolamide	€0.12		IQVIA [31], Ministère des Affaires Sociales et de la Santé [32]
Brinzolamide + brimonidine	€0.28		IQVIA [31], Ministère des Affaires Sociales et de la Santé [32]
Dorzolamide	€0.14		IQVIA [31], Ministère des Affaires Sociales et de la Santé [32]
Timolol	€0.18		IQVIA [31], Ministère des Affaires Sociales et de la Santé [32]
Timolol + dorzolamide	€0.20		IQVIA [31], Ministère des Affaires Sociales et de la Santé [32]
**OSD therapies** ^e^			
Cyclosporin	€3.67		IQVIA [31], Ministère des Affaires Sociales et de la Santé [32]
Preservative-free lubricant	€0.27		IQVIA [31], Ministère des Affaires Sociales et de la Santé [32]
**Healthcare procedures** ^**f**^			
Breakup time test	€19.20		Sécurité Sociale [33] (elaborated on: Tarif Secteur 1; code: 02.01.06.03 BBQP001)
	(€15.44; €22.96)	
Diurnal curve of intraocular pressure measurement	€41.66 (€33.49; €49.83)		Sécurité Sociale [33] (elaborated on: Tarif Secteur 1; code: 02.01.06.01 BHQP001)
Fluorescein test	€19.20		Sécurité Sociale [33] (elaborated on: Tarif Secteur 1; code: 02.01.06.03 BBQP001)
	(€15.44; €22.96)	
Gonioscopy	€17.28 (€13.89; €20.67)		Sécurité Sociale [33] (Tarif Secteur 1; code: 02.01.06.01 BHQP002)
Lissamine test	€19.20		Sécurité Sociale [33] (elaborated on: Tarif Secteur 1; code: 02.01.06.03 BBQP001)
	(€15.44; €22.96)		
Optical coherence tomography retinal nerve fiber layer	€56.54 (€45.46; €67.62)		Sécurité Sociale [33] (Tarif Secteur 1; code: 02.01.05 BZQK001)
Retinal nerve fiber thickness assessment	€26.78 (€21.53; €32.03)		Sécurité Sociale [33] (Tarif Secteur 1; code: 02.01.04 BGQP009)
Schirmer test	€19.20		Sécurité Sociale [33] (Tarif Secteur 1; code: 02.01.06.03 BBQP001)
	(€15.44; €22.96)	
Slit lamp examination	€28.29 (€24.92; €37.06)		Sécurité Sociale [33] (Tarif Secteur 1; code: 02.01.06.03 BGQP002)
Tonometry	€41.66 (€11.21; € 16.67)		Sécurité Sociale [33] (Tarif Secteur 1; code: 02.01.06.01 BHQP001)
Visual field test	€39.43		Sécurité Sociale [33] (Tarif Secteur 1; code: 02.01.06.02 BLQP004)
	(€31.70; € 47.16)	
**Specialist visits**			
Ophthalmologist	€25.32 (€20.36; €30.28)		Sécurité Sociale [33] (Tarif Secteur 1; code: 02.01.06.02 BLQP010)
**Utility and disutility values** ^**g**^	**STN1013001** ^**h**^	**Latanoprost** ^**1**^	
OAG/OHT stage 0	0.900 (0.885; 0.915)	0.900 (0.884; 0.915)	van Gestel et al. [29]
OAG/OHT stage 1	0.897 (0.880; 0.913)	0.890	Experts' opinion
		(0.872; 0.908)	
OAG/OHT stage 2	0.879 (0.860; 0.896)	0.866 (0.845; 0.885)	Experts' opinion
OAG/OHT stage 3	0.862 (0.840; 0.883)	0.849 (0.825; 0.871)	Experts' opinion
OAG/OHT stage 4	0.825 (0.795; 0.853)	0.808 (0.776; 0.838)	Experts' opinion
OAG/OHT stage 5	0.790 (0.750; 0.828)	0.790 (0.748; 0.829)	van Gestel et al. [29],
OSD-related disutility	−0.120	−0.120	Canadian Agency for drugs and Technology in Health [30]
	(−0.231; −0.045)	(−0.231; −0.045)	
Death	0.000 (−)	0.000 (−)	Drummond et al. [15], Neumann et al. [16]

^a^95% CI was calculated assuming a normal probability distribution [14,34].^b^95% CI was not calculated for the unit cost of drugs since they are exogenous variables [34].^c^ Medications refer to STN1013001 and Latanoprost only. ^d^Cost per diem calculated on ex-factory price. ^e^Add-on therapies prescribed in addition to STN1013001 or Latanoprost due to poor IOP control. ^f^Being not funded by French health system, LipiView test was not included in cost calculation. ^g^95% CI for utility and disutility values was calculated assuming a beta and a gamma probability distribution, respectively [14]. ^h^Number of observations per OAG/OHT stage (female %): 0 = 1560 (50.00%%); 1 = 1280 (50.00%); 2 = 1280 (50.00%); 3 = 1000 (50.00%); 4 = 650 (50.00%); and 5 = 415 (50.00%). ^i^Number of observations per OAG/OHT stage: 0 = 1460 (50.00%); 1 = 1160 (57.43%); 2 = 1150 (50.00%); 3 = 930 (50.00%); 4 = 61 (50.00%); and 5 = 390 (50.00%). CI = confidence interval; IOP = intraocular pressure; OAG/OHT = open-angle glaucoma/ocular hypertension; and OSD = ocular surface disease. Bold fonts were used to define different sets of items that were used to populate the Markov model.

**Table 2:** Base case analysis–results–costs per patient and cost-utility analysis (€2020).

Items	STN1013001 (%)	Latanoprost (%)	Difference (%) [95% CI]^a,b^
French health system viewpoint			
Cost			
Diagnosis	€191.60 (8.16)	€192.75 (8.24)	−€1.15 (14.63) [−€32.62; €30.80]
Medications	€341.28 (14.54)	€241.81 (10.33)	€99.47 (−1070.48) [€95.04; €104.53]
Add-on therapies and follow-up	€1337.59 (56.97)	€1339.40 (57.23)	−€1.81 (22.14) [−€146.76; €143.26]
OSD management	€477.27 (20.33)	€566.38 (24.20)	−€89.11 (1133.72) [−€160.16; −€19.73]
**Overall**	**€2347.74 (100.00)**	**€2340.34 (100.00)**	**€7.39 (100) [**−**€159.39; €166.60]**

**LYS and QALYs**			
**LYS**	4.228	4.223	0.005 [−0.013; 0.023]
**QALYs**	2.539	2.191	0.348 [0.285; 0.408]

**Cost-utility analysis**			
**Incremental costs (ΔC)**	€7.39		
**Incremental QALYs (ΔQALYs)**	0.348		
**ICUR (ΔC/ΔQALYs)**	**€21.26**		

^a^(STN1013001–Latanoprost). ^b^95% CI was calculated via the percentile method [34]. CI = confidence interval; ICUR = incremental cost-utility ratio; LYS = life year saved; OAG/OHT = open-angle glaucoma/ocular hypertension; OSD = ocular surface disease; and QALYs = quality-adjusted life years. Bold fonts were used to highlight some results.

## Data Availability

Data are provided in the Supplementary Information files submitted alongside the manuscript.

## Supplementary Materials

Supplementary Materials
*SText*. Probabilistic sensitivity analysis: essential glossary *Figure S1*. Base case analysis-results-mean cost per patient per OAG/OHT stage^a,b^. *Figure S2*. Base case analysis-results-mean QALYs per patient per OAG/OHT stage^a,b^. *Table S1*. Base case analysis-methods-OAG/OHT staging^a^. *Table S2*. Base case analysis-methods-transition probability matrix (95% CI)^a^. *Table S3*. Base case analysis-results-OAG/OHT patients' age (range). *Table S4*. Base case analysis-results-mean number (SD) of OAG/OHT notional patients in each Markov state during a 5-year time horizon. *Table S5*. Base case analysis-results-adherence probabilities to OAG/OHT medications (95% CI)^a,b^. Table S6. Base case analysis-results-healthcare resource average consumption (95% CI)^a^-diagnosis. *Table S7*. Base case analysis-results-healthcare resource average consumption-management and follow-up-I-add-on therapies and drugs (range)a. *Table S8*. Base case analysis-results-healthcare resource average consumption (95% CI)^a^-management and follow-up-II-healthcare procedures and specialist visits. *Table S9*. Base case analysis-results-healthcare resource average consumption-OSD management-I-drugs^a,b^. *Table S10*. Base case analysis-results-healthcare resource average consumption (95% CI)^a,b^-OSD management-II-healthcare procedures and specialist visits.
