# Supplementary material for: Cost-Utility Analysis of STN1013001, a Latanoprost Cationic Emulsion, versus Other Latanoprost Formulations (Latanoprost) in Open-Angle Glaucoma or Ocular Hypertension and Ocular Surface Disease in France
Source: J Ophthalmol. 2022 Apr 29;2022:3837471. doi: 10.1155/2022/3837471 (PMC9076337; doi:10.1155/2022/3837471)
Supplement: Supplementary Materials — SText. Probabilistic sensitivity analysis: essential glossary Figure S1. Base case analysis-results-mean cost per patient per OAG/OHT stagea,b. Figure S2. Base case analysis-results-mean QALYs per patient per OAG/OHT stagea,b. Table S1. Base case analysis-methods-OAG/OHT staginga. Table S2. Base case analysis-methods-transition probability matrix (95% CI)a. Table S3. Base case analysis-results-OAG/OHT patients' age (range). Table S4. Base case analysis-results-mean number (SD) of OAG/OHT notional patients in each Markov state during a 5-year time horizon. Table S5. Base case analysis-results-adherence probabilities to OAG/OHT medications (95% CI)a,b. Table S6. Base case analysis-results-healthcare resource average consumption (95% CI)a-diagnosis. Table S7. Base case analysis-results-healthcare resource average consumption-management and follow-up-I-add-on therapies and drugs (range)a. Table S8. Base case analysis-results-healthcare resource average consumption (95% CI)a-management and follow-up-II-healthcare procedures and specialist visits. Table S9. Base case analysis-results-healthcare resource average consumption-OSD management-I-drugsa,b. Table S10. Base case analysis-results-healthcare resource average consumption (95% CI)a,b-OSD management-II-healthcare procedures and specialist visits. [file 3837471.f1.zip › Rev_3837471.f1/Journal_of_Ophthalmology_Fig.S2.pdf]

Figure S2. Base case analysis–results–mean QALYs per patient per OAG/OHT stage[a,b]

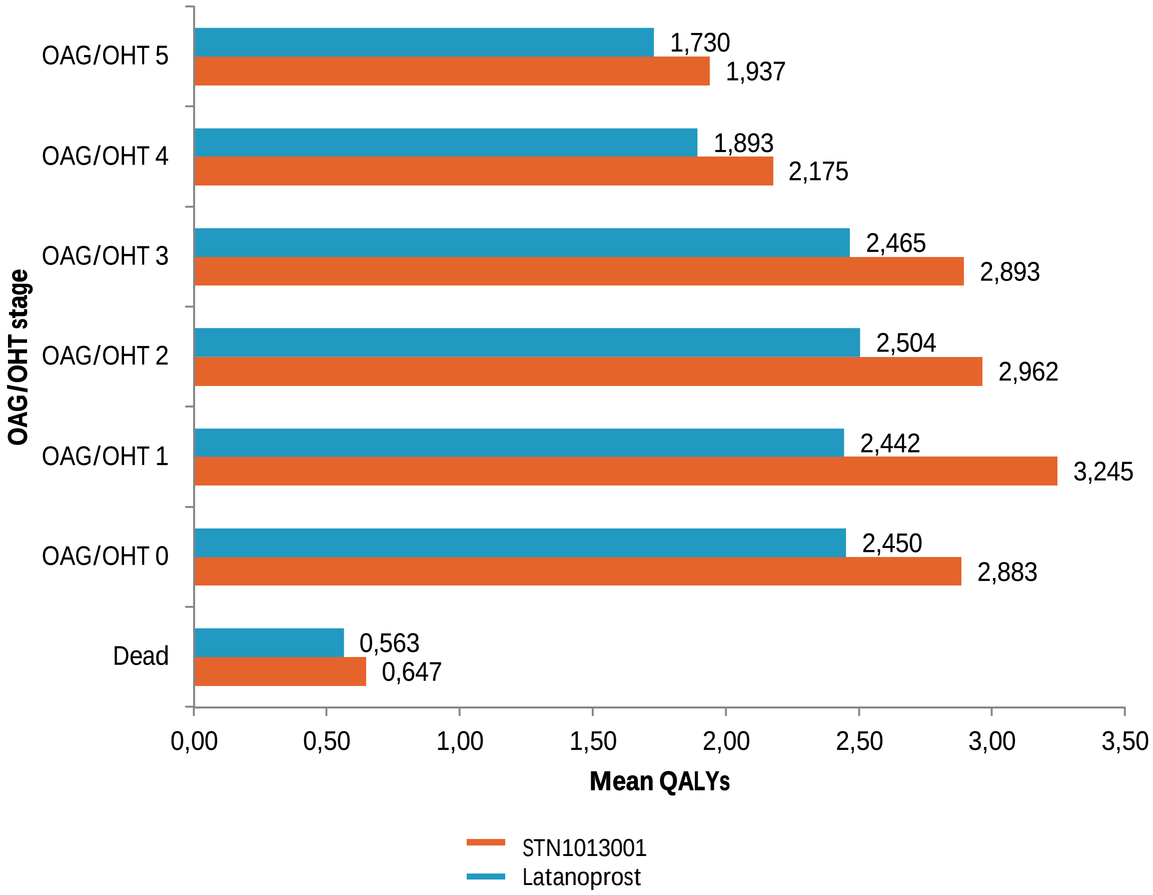

[a] Notional patients on STN1013001 totaled higher ΔQALYs in all the Markov model health states: Dead OAG/OHT patients (ΔQALYs: 0.084; 95% CI: 0.062; 0.108), OAG/OHT stage 0 (ΔQALYs: 0.432; 95% CI: 0.340; 0.525), OAG/OHT stage 1 (ΔQALYs: 0.803; 95% CI: 0.716; 0.892), OAG/OHT stage 2 (ΔQALYs: 0.459; 95% CI: 0.348; 0.566), OAG/OHT stage 3 (ΔQALYs: 0.428; 95% CI: 0.299; 0.557), OAG/OHT stage 4 (ΔQALYs: 0.282; 95% CI: 0.146; 0.419), and OAG/OHT stage 5 (ΔQALYs: 0.208; 95% CI: 0.036; 0.378).
[b] 95% CI was calculated via the percentile method [34].
CI = confidence interval; ΔC = incremental cost; OAG/OHT = open-angle glaucoma/ocular hypertension.
