# Supplementary material for: Cost-Utility Analysis of STN1013001, a Latanoprost Cationic Emulsion, versus Other Latanoprost Formulations (Latanoprost) in Open-Angle Glaucoma or Ocular Hypertension and Ocular Surface Disease in France
Source: J Ophthalmol. 2022 Apr 29;2022:3837471. doi: 10.1155/2022/3837471 (PMC9076337; doi:10.1155/2022/3837471)
Supplement: Supplementary Materials — SText. Probabilistic sensitivity analysis: essential glossary Figure S1. Base case analysis-results-mean cost per patient per OAG/OHT stagea,b. Figure S2. Base case analysis-results-mean QALYs per patient per OAG/OHT stagea,b. Table S1. Base case analysis-methods-OAG/OHT staginga. Table S2. Base case analysis-methods-transition probability matrix (95% CI)a. Table S3. Base case analysis-results-OAG/OHT patients' age (range). Table S4. Base case analysis-results-mean number (SD) of OAG/OHT notional patients in each Markov state during a 5-year time horizon. Table S5. Base case analysis-results-adherence probabilities to OAG/OHT medications (95% CI)a,b. Table S6. Base case analysis-results-healthcare resource average consumption (95% CI)a-diagnosis. Table S7. Base case analysis-results-healthcare resource average consumption-management and follow-up-I-add-on therapies and drugs (range)a. Table S8. Base case analysis-results-healthcare resource average consumption (95% CI)a-management and follow-up-II-healthcare procedures and specialist visits. Table S9. Base case analysis-results-healthcare resource average consumption-OSD management-I-drugsa,b. Table S10. Base case analysis-results-healthcare resource average consumption (95% CI)a,b-OSD management-II-healthcare procedures and specialist visits. [file 3837471.f1.zip › Rev_3837471.f1/Rev_Supporting_Information_SText_Journal_of_Ophthalmology(1).docx]

***SText*. Probabilistic sensitivity analysis - essential glossary**

**Definition S1. Cost-effectiveness plane (CEP):** CEP [40] is a cartesian plane (X-axis: incremental effectiveness; Y-axis: incremental cost), divided in four sectors labelled counterclock-wise with the compass headings, starting from the right upper one. Assuming the most trivial case of two healthcare programmes under comparison, the four sectors of the CEP lead the healthcare decision-maker to the following choices:

North-East (NE) sector (incremental cost [ΔC]>0;incremental effectiveness[ΔE]>0): the healthcare programme of interest shows higher costs and higher effectiveness that the comparator. In this instance, incremental cost-effectiveness ratio (ICER) calculation and subsequent contrast against a jurisdiction-specific threshold value is relevant to decide whether or not the healthcare programme is affordable (i.e., cost-effective) for the third payer;

North-West (NW) sector (ΔC>0;ΔE<0): the healthcare programme under investigation is said to be strongly dominated, as it is more costly and less effective than the comparator. Strongly dominated healthcare programmes should be always rejected, as their funding implies wasting limited resources. As such, ICER calculation is not necessary to support the decision-making process;

South-West (SW) sector (ΔC<0;ΔE<0): this is probably the most critical sector of the CEP. Funding a healthcare programme less costly but also less effective than the comparator is often mandatory under heavy budget constraints. In this instance, ICER calculation and subsequent contrast against a jurisdiction-specific threshold value helps healthcare decision-maker to assess the trade-off between cost-saving and decreased effectiveness of the healthcare programme under investigation;

South-East (SE) sector (ΔC<0;ΔE>0): the healthcare programme under investigation is said to be strongly dominant, being at the same time less costly and more effective than the comparator. Strongly dominant healthcare programmes should be always funded, as their higher effectiveness is coupled with a remarkable economic efficiency. As such, ICER calculation is not necessary to support the decision-making process.

The description of the different sectors of the CEP holds for Incremental Cost-Utility Ratio (ICUR), too. It is simply a matter of replacing incremental effectiveness and ICER with incremental QALYs (ΔQALYs) and ICUR.

**Definition S2. Net monetary benefit (NMB):** The ICUR inequality is:

$$ICUR<\lambda$$

or, put differently:

$$\frac{\Delta C}{\Delta QALYs}<\lambda$$

where *λ* is a willingness to pay (WTP) per ΔQALY gained set by a given jurisdiction.

The NMB is an algebraic manipulation of the terms included in ICUR computation [14-16, 34, 39, 41, 42]:

(*NMB_A_ – NMB_B_*) = [(*λ* **QALYs_A_*) – *Cost_A_*] – [(*λ* **QALYs_B_*) – Cost_B_]

Therefore, the incremental NMB (ΔNMB) is a different way of representing the numerator and denominator of the ICUR (*ΔC and ΔQALYs*):

*ΔNMB* = [*λ* *(*QALYs_A_* – *QALYs_B_*)] – (*Cost_A_* – *Cost_B_*)

which is equivalent to:

*ΔNMB* = [(*λ* **ΔQALYs*) – *ΔC*]

The reversed sign of ICUR inequality indicates the condition for the healthcare technology under investigation to be cost-effective:

$$\Delta NMB>0$$

**Definition S3. Cost-effectiveness acceptability curve** **(CEAC):** CEAC is a plot that shows the uncertainty surrounding the base case estimate of the ICUR.

A set of threshold values (say, from €0 to €200,000) that represent the healthcare policy makers’ WTP for ΔQALYs are reported on the x-axis, and the probability for the healthcare technology under investigation to be cost-effective is reported on the y-axis.

Healthcare policy-makers can obtain the probability for the healthcare program under investigation to be cost-effective for a given WTP by looking across from the y-axis to the CEAC [14-16, 34, 41, 43, 44].

**Definition S4. Cost-effectiveness acceptability frontier (CEAF):** elaborating on the CEAC, the CEAF is a graphical method that represents the probability that the healthcare program with the highest average NMB is also cost-effective [14-16, 34, 43, 44].
