# Supplementary material for: Cost-Utility Analysis of STN1013001, a Latanoprost Cationic Emulsion, versus Other Latanoprost Formulations (Latanoprost) in Open-Angle Glaucoma or Ocular Hypertension and Ocular Surface Disease in France
Source: J Ophthalmol. 2022 Apr 29;2022:3837471. doi: 10.1155/2022/3837471 (PMC9076337; doi:10.1155/2022/3837471)
Supplement: Supplementary Materials — SText. Probabilistic sensitivity analysis: essential glossary Figure S1. Base case analysis-results-mean cost per patient per OAG/OHT stagea,b. Figure S2. Base case analysis-results-mean QALYs per patient per OAG/OHT stagea,b. Table S1. Base case analysis-methods-OAG/OHT staginga. Table S2. Base case analysis-methods-transition probability matrix (95% CI)a. Table S3. Base case analysis-results-OAG/OHT patients' age (range). Table S4. Base case analysis-results-mean number (SD) of OAG/OHT notional patients in each Markov state during a 5-year time horizon. Table S5. Base case analysis-results-adherence probabilities to OAG/OHT medications (95% CI)a,b. Table S6. Base case analysis-results-healthcare resource average consumption (95% CI)a-diagnosis. Table S7. Base case analysis-results-healthcare resource average consumption-management and follow-up-I-add-on therapies and drugs (range)a. Table S8. Base case analysis-results-healthcare resource average consumption (95% CI)a-management and follow-up-II-healthcare procedures and specialist visits. Table S9. Base case analysis-results-healthcare resource average consumption-OSD management-I-drugsa,b. Table S10. Base case analysis-results-healthcare resource average consumption (95% CI)a,b-OSD management-II-healthcare procedures and specialist visits. [file 3837471.f1.zip › Rev_3837471.f1/Rev_Supporting_Information_Table_S1_Journal_of_Ophthalmology(1).docx]

***Table S1.*** Base case analysis–methods–OAG/OHT staging^a^

| OAG/OHT stage | Description | MD score^b^ | Probability plot  (pattern deviation) ^b^ |
| --- | --- | --- | --- |
| OAG/OHT stage 0 | Ocular hypertension | 0.00 | Does not meet stage 1 criteria |
| OAG/OHT stage 1 | Early glaucoma | -0.01 to -6.00 | Points below 5% >3 contiguous and >1 of the points is below 1% |
| OAG/OHT stage 2 | Moderate glaucoma | -6.01 to -12.00 | Points below 5%: 19–36 and points below 1%: 12–18 |
| OAG/OHT stage 3 | Advanced glaucoma | -12.01 to -20.00 | Points below 5%: 37–55 and points below 1%: 19–36 |
| OAG/OHT stage 4 | Severe glaucoma | -20.01 or worse | Points below 5%: 56–74 and points below 1%: 37–74 |
| OAG/OHT stage 5 | End-stage/blindness | No threshold | Not possible |

^a^ Adapted from [5, 6].

^b^ Both indexes to be considered for diagnostic purposes.

MD=mean defect or mean deviation; OAG/OHT=primary open-angle glaucoma/ocular hypertension.
