# Supplementary material for: Cost-Utility Analysis of STN1013001, a Latanoprost Cationic Emulsion, versus Other Latanoprost Formulations (Latanoprost) in Open-Angle Glaucoma or Ocular Hypertension and Ocular Surface Disease in France
Source: J Ophthalmol. 2022 Apr 29;2022:3837471. doi: 10.1155/2022/3837471 (PMC9076337; doi:10.1155/2022/3837471)
Supplement: Supplementary Materials — SText. Probabilistic sensitivity analysis: essential glossary Figure S1. Base case analysis-results-mean cost per patient per OAG/OHT stagea,b. Figure S2. Base case analysis-results-mean QALYs per patient per OAG/OHT stagea,b. Table S1. Base case analysis-methods-OAG/OHT staginga. Table S2. Base case analysis-methods-transition probability matrix (95% CI)a. Table S3. Base case analysis-results-OAG/OHT patients' age (range). Table S4. Base case analysis-results-mean number (SD) of OAG/OHT notional patients in each Markov state during a 5-year time horizon. Table S5. Base case analysis-results-adherence probabilities to OAG/OHT medications (95% CI)a,b. Table S6. Base case analysis-results-healthcare resource average consumption (95% CI)a-diagnosis. Table S7. Base case analysis-results-healthcare resource average consumption-management and follow-up-I-add-on therapies and drugs (range)a. Table S8. Base case analysis-results-healthcare resource average consumption (95% CI)a-management and follow-up-II-healthcare procedures and specialist visits. Table S9. Base case analysis-results-healthcare resource average consumption-OSD management-I-drugsa,b. Table S10. Base case analysis-results-healthcare resource average consumption (95% CI)a,b-OSD management-II-healthcare procedures and specialist visits. [file 3837471.f1.zip › Rev_3837471.f1/Rev_Supporting_Information_Table_S10_Journal_of_Ophthalmology(1).docx]

***Table S10*.** Base case analysis–results–healthcare resource average consumption (95% CI)^a,b^–OSD management–II–healthcare procedures and specialist visits

| Cost items | STN1013001 | % targeted patients |  | Latanoprost | % targeted patients | Δ% targeted patients^b,c^ |
| --- | --- | --- | --- | --- | --- | --- |
| OAG/OHT stage 0 | N=1560 |  |  | N=1460 |  |  |
| Healthcare procedures | | | | | | |
| Breakup time test | 0.71 (0.46; 1.01) | 49.36% |  | 0.68 (0.44; 0.98) | 54.79% | -5.44% (-9.06%; -1.85%) |
| Fluorescin test | 0.38 (0.33; 0.45) | 25.64% |  | 0.41 (0.35; 0.48) | 27.40% | -1.76% (-8.11%; 1.37%) |
| Lissamin test | 0.13 (0.08; 0.18) | 1.92% |  | 0.14 (0.09; 0.20) | 2.74% | -0.82% (-1.89%; 0.25%) |
| Schirmer test | 1.09 (0.86; 1.35) | 11.54% |  | 1.10 (0.87; 1.32) | 20.55% | -9.01% (-11.67%; -6.41%) |
| Slit lamp examination | 1.87 (1.40; 2.41) | 100.00% |  | 1.86 (1.41; 2.38) | 100.00% | - |
| Specialist visits | | | | | | |
| Ophthalmologist | 2.00 (1.53; 2.53) | 100.00% |  | 2.00 (1.55; 2.51) | 100.00% | - |
| OAG/OHT stage 1 | N=1280 |  |  | N=1160 |  |  |
| Healthcare procedures | | | | | | |
| Breakup time test | 0.55 (0.35; 0.78) | 38.28% |  | 0.52 (0.33; 0.74) | 41.38% | -3.10% (-6.99%; 0.76%) |
| Fluorescin test | 0.63 (0.53; 0.73) | 39.06% |  | 0.41 (0.31; 0.53) | 27.40% | 11.67% (7.93%; 15.45%) |
| Lissamin test | 0.13 (0.07; 0.20) | 1.92% |  | 0.14 (0.08; 0.20) | 2.74% | -0.82% (-2.04%; 0.39%) |
| Schirmer test | 1.17 (1.00; 1.35) | 12.50% |  | 1.10 (0.94; 1.27) | 20.55% | -8.05% (-11.02%; -5.12%) |
| Slit lamp examination | 1.84 (1.50; 2.22) | 100.00% |  | 1.84 (1.53; 2.19) | 100.00% | - |
| Specialist visits | | | | | | |
| Ophthalmologist | 2.00 (1.67; 2.36) | 100.00% |  | 2.00 (1.70; 2.33) | 100.00% | - |
| OAG/OHT stage 2 | N=1280 |  |  | N=1150 |  |  |
| Healthcare procedures | | | | | | |
| Breakup time test | 0.55 (0.35; 0.78) | 38.28% |  | 0.68 (0.50; 0.90) | 54.79% | -16.51% (-20.39%; -12.70%) |
| Fluorescin test | 0.61 (0.51; 0.72) | 37.50% |  | 0.41 (0.30; 0.53) | 27.40% | 10.10% (6.45%; 13.80%) |
| Lissamin test | 0.13 (0.07; 0.20) | 2.56% |  | 0.14 (0.08; 0.20) | 3.42% | -0.86% (-2.23%; 0.51%) |
| Schirmer test | 1.16 (0.99; 1.34) | 15.31% |  | 1.10 (0.93; 1.27) | 22.60% | -7.29% (-10.35%; -4.23%) |
| Slit lamp examination | 1.86 (1.52; 2.23) | 100.00% |  | 1.87 (1.55; 2.22) | 100.00% | - |
| Specialist visits | | | | | | |
| Ophthalmologist | 2.00 (1.67;2.36) | 100.00% |  | 1.99 (1.68;2.31) | 100.00% | - |
| OAG/OHT stage 3 | N=1000 |  |  | N=930 |  |  |
| Healthcare procedures | | | | | | |
| Breakup time test | 0.55 (0.36; 0.79) | 44.00% |  | 0.54 (0.35; 0.77) | 53.76% | -9.76% (-14.75%; -4.66%) |
| Fluorescin test | 0.55 (0.46; 0.64) | 35.00% |  | 0.57 (0.48; 0.67) | 35.48% | -0.48% (-4.72%; 3.69%) |
| Lissamin test | 0.13 (0.08; 0.19) | 2.56% |  | 0.14 (0.08; 0.20) | 3.42% | -0.86% (-2.64%; 0.78%) |
| Schirmer test | 1.10 (0.93; 1.28) | 20.00% |  | 1.11 (0.94; 1.29) | 28.23% | -8.23% (-12.01; -4.41%) |
| Slit lamp examination | 1.95 (1.61; 2.32) | 100.00% |  | 1.97 (1.64; 2.32) | 100.00% | - |
| Specialist visits | | | | | | |
| Ophthalmologist | 2.10 (1.78; 2.45) | 100.00% |  | 2.11 (1.80; 2.44) | 100.00% | - |
| OAG/OHT stage 4 | N=650 |  |  | N=610 |  |  |
| Healthcare procedures | | | | | | |
| Breakup time test | 0.40 (0.26; 0.57) | 32.00% |  | 0.41 (0.27; 0.59) | 40.98% | -8.98% (-14.29%; -3.72%) |
| Fluorescin test | 0.69 (0.58; 0.81) | 44.62% |  | 0.69 (0.58; 0.81) | 42.62% | 1.99% (-3.42%; 7.44%) |
| Lipiview test | 0.13 (0.06; 0.22) | 1.92% |  | 0.14 (0.08; 0.21) | 2.74% | -0.82% (-2.52%; 0.84%) |
| Lissamin test | 0.13 (0.06; 0.22) | 3.21% |  | 0.14 (0.08; 0.21) | 4.11% | -0.90% (-3.01%; 1.16%) |
| Schirmer test | 1.09 (0.97; 1.22) | 25.31% |  | 1.10 (0.97; 1.23) | 32.95% | -7.64% (-12.63%; -2.57%) |
| Slit lamp examination | 1.95 (1.74; 2.18) | 100.00% |  | 2.00 (1.78; 2.24) | 100.00% | - |
| Specialist visits | | | | | | |
| Ophthalmologist | 3.00 (2.70; 3.31) | 100.00% |  | 3.00 (2.70; 3.32) | 100.00% | - |
| OAG/OHT stage 5 | N=415 |  |  | N=390 |  |  |
| Healthcare procedures | | | | | | |
| Breakup time test | 0.23 (0.15; 0.33) | 20.60% |  | 0.23 (0.15; 0.33) | 23.08% | -2.47% (-8.14%; 3.17%) |
| Fluorescin test | 0.82 (0.69; 0.96) | 53.01% |  | 0.82 (0.69; 0.96) | 51.28% | 1.73% (-5.14%; 8.59%) |
| Lipiview test | 0.13 (0.05; 0.24) | 1.92% |  | 0.14 (0.07; 0.23) | 2.74% | 0.82 (-3.01%; 1.26%) |
| Lissamin test | 0.13 (0.06; 0.22) | 3.21% |  | 0.14 (0.07; 0.23) | 4.11% | -0.90% (-3.48%; 1.70%) |
| Schirmer test | 1.05 (0.95; 1.16) | 27.35% |  | 1.05 (0.94; 1.16) | 36.92% | -9.57% (-15.93%; -3.11%) |
| Slit lamp examination | 2.00 (1.83; 2.18) | 100.00% |  | 2.05 (1.86; 2.25) | 100.00% | - |
| Specialist visits | | | | | | |
| Ophthalmologist | 3.00 (2.87; 3.14) | 100.00% |  | 3.00 (2.88; 3.12) | 100.00% | - |

^a^Unless otherwise specified, 95% CI was calculated assuming a Gamma probability distribution [14, 34].

^b^(STN1013001 – Latanoprost).

^c^95% CI was calculated via the percentile method [34].

CI=confidence interval; N=number of observations OAG/OHT=open-angle glaucoma/ocular hypertension; OSD=ocular surface disease.
