# Supplementary material for: Cost-Utility Analysis of STN1013001, a Latanoprost Cationic Emulsion, versus Other Latanoprost Formulations (Latanoprost) in Open-Angle Glaucoma or Ocular Hypertension and Ocular Surface Disease in France
Source: J Ophthalmol. 2022 Apr 29;2022:3837471. doi: 10.1155/2022/3837471 (PMC9076337; doi:10.1155/2022/3837471)
Supplement: Supplementary Materials — SText. Probabilistic sensitivity analysis: essential glossary Figure S1. Base case analysis-results-mean cost per patient per OAG/OHT stagea,b. Figure S2. Base case analysis-results-mean QALYs per patient per OAG/OHT stagea,b. Table S1. Base case analysis-methods-OAG/OHT staginga. Table S2. Base case analysis-methods-transition probability matrix (95% CI)a. Table S3. Base case analysis-results-OAG/OHT patients' age (range). Table S4. Base case analysis-results-mean number (SD) of OAG/OHT notional patients in each Markov state during a 5-year time horizon. Table S5. Base case analysis-results-adherence probabilities to OAG/OHT medications (95% CI)a,b. Table S6. Base case analysis-results-healthcare resource average consumption (95% CI)a-diagnosis. Table S7. Base case analysis-results-healthcare resource average consumption-management and follow-up-I-add-on therapies and drugs (range)a. Table S8. Base case analysis-results-healthcare resource average consumption (95% CI)a-management and follow-up-II-healthcare procedures and specialist visits. Table S9. Base case analysis-results-healthcare resource average consumption-OSD management-I-drugsa,b. Table S10. Base case analysis-results-healthcare resource average consumption (95% CI)a,b-OSD management-II-healthcare procedures and specialist visits. [file 3837471.f1.zip › Rev_3837471.f1/Rev_Supporting_Information_Table_S2_Journal_of_Ophthalmology(1).docx]

## *Table S2*. Base case analysis–methods–transition probability matrix (95% CI)^a^

| From OAG/OHT stage 0 to | OAG/OHT stage 0^b^ | OAG/OHT  stage 1^b^ | OAG/OHT  stage 2^b^ | OAG/OHT  stage 3^b^ | OAG/OHT  stage 4^b^ | OAG/OHT  stage 5^b^ | Death^c,d^ |
| --- | --- | --- | --- | --- | --- | --- | --- |
| STN1013001 (N=1560) | | | | | | |  |
| Point estimates | (1 - other probabilities in the same row) | 0.065  (0.034; 0.104) | 0.048  (0.022; 0.84) | 0.006  (0.0002; 0.023) | 0.000  (-) | 0.000  (-) | 0.039  (0.037; 0.040) |
| Latanoprost (N=1460) | | | | | | |  |
| Point estimates | (1 - the other probabilities in the same row) | 0.075  (0.043; 0.116) | 0.049  (0.023; 0.085) | 0.010  (0.010; 0.010) | 0.000  (-) | 0.000  (-) | 0.039  (0.037; 0.040) |
| From OAG/OHT stage 1 to |  | OAG/OHT  stage 1^b^ | OAG/OHT  stage 2^b^ | OAG/OHT  stage 3^b^ | OAG/OHT  stage 4^b^ | OAG/OHT  stage 5^b^ | Death^c,d^ |
| STN1013001 (N=1280) | | | | | | |  |
| Point estimates |  | (1 - the other probabilities in the same row) | 0.100  (0.063; 0.145) | 0.047  (0.021; 0.082) | 0.012  (0.012; 0.012) | 0.000  (-) | 0.053  (0.051; 0.054) |
| Latanoprost (N=1160) | | | | | | |  |
| Point estimates |  | (1 - the other probabilities in the same row) | 0.103  (0.064; 0.150) | 0.061  (0.030; 0.101) | 0.000  (-) | 0.000  (-) | 0.053  (0.051; 0.054) |
| From OAG/OHT stage 2 to |  |  | OAG/OHT  stage 2^b^ | OAG/OHT  stage 3^b^ | OAG/OHT  stage 4^b^ | OAG/OHT  stage 5^b^ | Death^c,d^ |
| STN1013001 (N=1280) | | | | | | |  |
| Point estimates |  |  | (1 - the other probabilities in the same row) | 0.158  (0.119;  0.201) | 0.082  (0.052;  0.119) | 0.000  (-) | 0.057 (0.056; 0.059) |
| Latanoprost (N=1150) | | | | | | |  |
| Point estimates |  |  | (1 - the other probabilities in the same row) | 0.162  (0.122;  0.207) | 0.085  (0.053;0.124) | 0.000  (-) | 0.062  (0.061; 0.064) |
| From OAG/OHT stage 3 to |  |  |  | OAG/OHT  stage 3^b^ | OAG/OHT  stage 4^b^ | OAG/OHT  stage 5^b^ | Death^c,d^ |
| STN1013001 (N=1000) | | | | | | |  |
| Point estimates |  |  |  | (1 - the other probabilities in the same row) | 0.188  (0.144; 0.236) | 0.085  (0.052; 0.124) | 0.079  (0.077; 0.081) |
| Latanoprost (N=930) | | | | | | |  |
| Point estimates |  |  |  | (1 - the other probabilities in the same row) | 0.187  (0.142; 0.237) | 0.091  (0.056; 0.134) | 0.079  (0.077; 0.081) |
| From OAG/OHT stage 4 to |  |  |  |  | OAG/OHT  stage 4^b^ | OAG/OHT  stage 5^b^ | Death^c,d^ |
| STN1013001 (N=650) | | | | | | |  |
| Point estimates |  |  |  |  | (1 - the other probabilities in the same row) | 0.284  (0.221; 0.351) | 0.115  (0.113; 0.117) |
| Latanoprost (N=610) | | | | | | |  |
| Point estimates |  |  |  |  | (1 - the other probabilities in the same row) | 0.293  (0.229; 0.362) | 0.115  (0.113; 0.117) |
| From OAG/OHT stage 5 to |  |  |  |  |  | OAG/OHT  stage 5^b^ | Death^c,d^ |
| STN1013001 (N=415) | | | | | | |  |
| Point estimates |  |  |  |  |  | (1 - the other probabilities in the same row) | 0.163 (0.160; 0.165) |
| Latanoprost (N=390) | | | | | | |  |
| Point estimates |  |  |  |  |  | (1 - the other probabilities in the same row) | 0.163  (0.160; 0.165) |

^a^ 95% CI was calculated assuming a Dirichlet probability distribution [14, 34].

^b^ When stage probability was <0.02 or =1.000, in order to avoid impossible results in probabilistic sensitivity analysis, the Dirichlet distribution was not applied and the stage probability was set as fixed in base case and sensitivity analyses.

^c^ The reported average probability of all-cause mortality is gender and age-specific.

^d^ The 95% CI for all-cause mortality probability was calculated according to gender and age specific French life tables [24]. Due to 95% CI limited width around the point estimate, all-cause mortality probability was kept fixed in base case and sensitivity analyses.

CI=confidence interval; N=number of observations; OAG/OHT=primary open-angle glaucoma/ocular hypertension.
