# Supplementary material for: Cost-Utility Analysis of STN1013001, a Latanoprost Cationic Emulsion, versus Other Latanoprost Formulations (Latanoprost) in Open-Angle Glaucoma or Ocular Hypertension and Ocular Surface Disease in France
Source: J Ophthalmol. 2022 Apr 29;2022:3837471. doi: 10.1155/2022/3837471 (PMC9076337; doi:10.1155/2022/3837471)
Supplement: Supplementary Materials — SText. Probabilistic sensitivity analysis: essential glossary Figure S1. Base case analysis-results-mean cost per patient per OAG/OHT stagea,b. Figure S2. Base case analysis-results-mean QALYs per patient per OAG/OHT stagea,b. Table S1. Base case analysis-methods-OAG/OHT staginga. Table S2. Base case analysis-methods-transition probability matrix (95% CI)a. Table S3. Base case analysis-results-OAG/OHT patients' age (range). Table S4. Base case analysis-results-mean number (SD) of OAG/OHT notional patients in each Markov state during a 5-year time horizon. Table S5. Base case analysis-results-adherence probabilities to OAG/OHT medications (95% CI)a,b. Table S6. Base case analysis-results-healthcare resource average consumption (95% CI)a-diagnosis. Table S7. Base case analysis-results-healthcare resource average consumption-management and follow-up-I-add-on therapies and drugs (range)a. Table S8. Base case analysis-results-healthcare resource average consumption (95% CI)a-management and follow-up-II-healthcare procedures and specialist visits. Table S9. Base case analysis-results-healthcare resource average consumption-OSD management-I-drugsa,b. Table S10. Base case analysis-results-healthcare resource average consumption (95% CI)a,b-OSD management-II-healthcare procedures and specialist visits. [file 3837471.f1.zip › Rev_3837471.f1/Rev_Supporting_Information_Table_S3_Journal_of_Ophthalmology(1).docx]

***Table S3*.** Base case analysis–results–OAG/OHT patients’age (range)

| Age | STN1013001 |  | Latanoprost |  |
| --- | --- | --- | --- | --- |
| OAG/OHT stage 0 | N=1560 |  | N=1460 |  |
| Age (years) | 47.31 (45.00; 55.00) |  | 47.31 (45.00; 55.00) |  |
| OAG/OHT stage 1 | N=1280 |  | N=1160 |  |
| Age (years) | 51.02 (45.00; 60.00) |  | 51.02 (45.00; 60.00) |  |
| OAG/OHT stage 2 | N=1280 |  | N=1150 |  |
| Age (years) | 52.66 (45.00; 62.00) |  | 52.66 (45.00; 62.00) |  |
| OAG/OHT stage 3 | N=1000 |  | N=930 |  |
| Age (years) | 56.15 (50.00; 62.00) |  | 56.15 (50.00; 62.00) |  |
| OAG/OHT stage 4 | N=650 |  | N=610 |  |
| Age (years) | 61.77 (55.00; 65.00) |  | 61.77 (55.00; 65.00) |  |
| OAG/OHT stage 5 | N=415 |  | N=390 |  |
| Age (years) | 66.27 (60.00; 65.00) |  | 66.27 (60.00; 65.00) |  |

N=number of observations; OAG/OHT=open-angle glaucoma/ocular hypertension.
