# Supplementary material for: Cost-Utility Analysis of STN1013001, a Latanoprost Cationic Emulsion, versus Other Latanoprost Formulations (Latanoprost) in Open-Angle Glaucoma or Ocular Hypertension and Ocular Surface Disease in France
Source: J Ophthalmol. 2022 Apr 29;2022:3837471. doi: 10.1155/2022/3837471 (PMC9076337; doi:10.1155/2022/3837471)
Supplement: Supplementary Materials — SText. Probabilistic sensitivity analysis: essential glossary Figure S1. Base case analysis-results-mean cost per patient per OAG/OHT stagea,b. Figure S2. Base case analysis-results-mean QALYs per patient per OAG/OHT stagea,b. Table S1. Base case analysis-methods-OAG/OHT staginga. Table S2. Base case analysis-methods-transition probability matrix (95% CI)a. Table S3. Base case analysis-results-OAG/OHT patients' age (range). Table S4. Base case analysis-results-mean number (SD) of OAG/OHT notional patients in each Markov state during a 5-year time horizon. Table S5. Base case analysis-results-adherence probabilities to OAG/OHT medications (95% CI)a,b. Table S6. Base case analysis-results-healthcare resource average consumption (95% CI)a-diagnosis. Table S7. Base case analysis-results-healthcare resource average consumption-management and follow-up-I-add-on therapies and drugs (range)a. Table S8. Base case analysis-results-healthcare resource average consumption (95% CI)a-management and follow-up-II-healthcare procedures and specialist visits. Table S9. Base case analysis-results-healthcare resource average consumption-OSD management-I-drugsa,b. Table S10. Base case analysis-results-healthcare resource average consumption (95% CI)a,b-OSD management-II-healthcare procedures and specialist visits. [file 3837471.f1.zip › Rev_3837471.f1/Rev_Supporting_Information_Table_S4_Journal_of_Ophthalmology(1).docx]

***Table S4*.** Base case analysis–results–mean number (SD) of OAG/OHT notional patients in each Markov state during a 5-year time horizon

| Markov states | STN1013001 | % | Latanoprost | % |
| --- | --- | --- | --- | --- |
| OAG/OHT stage 0^a^ | 672 (215) | 67.21% | 649 (228) | 64.89% |
| OAG/OHT stage 1 | 95 (54) | 9.48% | 107 (60) | 10.72% |
| OAG/OHT stage 2 | 75 (44) | 7.47% | 76 (45) | 7.64% |
| OAG/OHT stage 3 | 27 (22) | 2.69% | 34 (27) | 3.37% |
| OAG/OHT stage 4 | 13 (13) | 1.27% | 13(14) | 1.30% |
| OAG/OHT stage 5 | 5 (7) | 0.49% | 6 (8) | 0.60% |
| Dead | 114 (81) | 11.38% | 115 (82) | 11.49% |
| Total | 1000 (-) | 100.00% | 1000 (-) | 100.00% |

^a^ OAG/OHT stage 0 is the Markov state when notional patients enter the model.

OAG/OHT=open-angle glaucoma/ocular hypertension; SD=standard deviation.
