# Supplementary material for: Cost-Utility Analysis of STN1013001, a Latanoprost Cationic Emulsion, versus Other Latanoprost Formulations (Latanoprost) in Open-Angle Glaucoma or Ocular Hypertension and Ocular Surface Disease in France
Source: J Ophthalmol. 2022 Apr 29;2022:3837471. doi: 10.1155/2022/3837471 (PMC9076337; doi:10.1155/2022/3837471)
Supplement: Supplementary Materials — SText. Probabilistic sensitivity analysis: essential glossary Figure S1. Base case analysis-results-mean cost per patient per OAG/OHT stagea,b. Figure S2. Base case analysis-results-mean QALYs per patient per OAG/OHT stagea,b. Table S1. Base case analysis-methods-OAG/OHT staginga. Table S2. Base case analysis-methods-transition probability matrix (95% CI)a. Table S3. Base case analysis-results-OAG/OHT patients' age (range). Table S4. Base case analysis-results-mean number (SD) of OAG/OHT notional patients in each Markov state during a 5-year time horizon. Table S5. Base case analysis-results-adherence probabilities to OAG/OHT medications (95% CI)a,b. Table S6. Base case analysis-results-healthcare resource average consumption (95% CI)a-diagnosis. Table S7. Base case analysis-results-healthcare resource average consumption-management and follow-up-I-add-on therapies and drugs (range)a. Table S8. Base case analysis-results-healthcare resource average consumption (95% CI)a-management and follow-up-II-healthcare procedures and specialist visits. Table S9. Base case analysis-results-healthcare resource average consumption-OSD management-I-drugsa,b. Table S10. Base case analysis-results-healthcare resource average consumption (95% CI)a,b-OSD management-II-healthcare procedures and specialist visits. [file 3837471.f1.zip › Rev_3837471.f1/Rev_Supporting_Information_Table_S5_Journal_of_Ophthalmology(1).docx]

***Table S5*.** Base case analysis–results–adherence probabilities to OAG/OHT medications (95% CI)^a,b^

| OAG/OHT stage | STN1013001 | Latanoprost | Difference^c^ |
| --- | --- | --- | --- |
| OAG/OHT stage 0 | N=1560 | N=1460 |  |
| Year 1 | 0.815 (0.796; 0.834) | 0.734 (0.711; 0.757) | 0.081 (0.052; 0.111) |
| Year 2 | 0.728 (0.706; 0.750) | 0.648 (0.623; 0.672) | 0.080 (0.048; 0.113) |
| Year 3 | 0.728 (0.706; 0.750) | 0.648 (0.623; 0.672) | 0.080 (0.047; 0.113) |
| Year 4 | 0.712 (0.689; 0.734) | 0.623 (0.598; 0.648) | 0.088 (0.055; 0.122) |
| Year 5 | 0. 635 (0.611; 0. 658) | 0.555 (0.529; 0.580) | 0.080 (0.045; 0.114) |
| OAG/OHT stage 1 | N=1280 | N=1160 |  |
| Year 1 | 0.809 (0.787; 0.830) | 0.734 (0.711; 0.757) | 0.073 (0.040; 0.106) |
| Year 2 | 0.741 (0.716; 0.764) | 0.648 (0.623; 0.672) | 0.071 (0.034; 0.108) |
| Year 3 | 0.729 (0.704; 0.753) | 0.648 (0.623; 0.672) | 0.078 (0.052; 0.114) |
| Year 4 | 0.715 (0.690; 0.7392) | 0.623 (0.598; 0.648) | 0.098 (0.060; 0.135) |
| Year 5 | 0.631 (0.604; 0.657) | 0.555 (0.529; 0.580) | 0.066 (0.027; 0.105) |
| OAG/OHT stage 2 | N=1280 | N=1150 |  |
| Year 1 | 0.823 (0.801; 0.843) | 0.774 (0.749; 0.798) | 0.049 (0.016; 0.080) |
| Year 2 | 0.780 (0.757; 0.803) | 0.707 (0.680; 0.732) | 0.074 (0.039; 0.108) |
| Year 3 | 0.775 (0.751; 0.797) | 0.680 (0.653; 0.707) | 0.094 (0.059; 0.130) |
| Year 4 | 0.754 (0.730; 0.777) | 0.665 (0.638; 0.692) | 0.089 (0.053; 0.125) |
| Year 5 | 0.688 (0.662; 0.713) | 0.600 (0.572; 0.628) | 0.088 (0.050; 0.125) |
| OAG/OHT stage 3 | N=1000 | N=930 |  |
| Year 1 | 0.815 (0.790; 0.838) | 0.768 (0.740; 0.794) | 0.047 (0.010; 0.083) |
| Year 2 | 0.770 (0.743; 0.796) | 0.696 (0.666; 0.725) | 0.074 (0.034; 0.113) |
| Year 3 | 0.760 (0.733; 0.786) | 0.685 (0.655; 0.715) | 0.075 (0.034; 0.114) |
| Year 4 | 0.738 (0.710; 0.764) | 0.657 (0.626; 0.687) | 0.081 (0.040; 0.120) |
| Year 5 | 0.728 (0.699; 0.755) | 0.646 (0.615; 0.677) | 0.081 (0.040; 0.123) |
| OAG/OHT stage 4 | N=650 | N=610 |  |
| Year 1 | 0.803 (0.772; 0.833) | 0.761 (0.726; 0.794) | 0.042 (-0.006; 0.089) |
| Year 2 | 0.785 (0.753; 0.816) | 0.744 (0.709; 0.778) | 0.041 (-0.006; 0.088) |
| Year 3 | 0.765 (0.732; 0.797) | 0.723 (0.687; 0.758) | 0.042 (-0.006; 0.090) |
| Year 4 | 0.742 (0.707; 0.774) | 0.693 (0.656; 0.729) | 0.048 (-0.003; 0.099) |
| Year 5 | 0.729 (0.694; 0.763) | 0.680 (0.643; 0.717) | 0.049 (-0.002; 0.100) |
| OAG/OHT stage 5 | N=415 | N=390 |  |
| Year 1 | 0.777 (0.736; 0.816) | 0.726 (0.680; 0.769) | 0.051 (-0.008; 0.111) |
| Year 2 | 0.753 (0.710; 0.793) | 0.710 (0.664; 0.754) | 0.043 (-0.019; 0.105) |
| Year 3 | 0.722 (0.678; 0.764) | 0.672 (0.624; 0.717) | 0.050(-0.014; 0.113) |
| Year 4 | 0.683 (0.638; 0.727) | 0.649 (0.601; 0.695) | 0.034(-0.030; 0.098) |
| Year 5 | 0.669 (0.623; 0.713) | 0.633 (0.585; 0.680) | 0.035(-0.028; 0.103) |

^a^ Medications refer to STN1013001 and Latanoprost only.

^b^ 95% CI was calculated assuming a Beta probability distribution [14, 34].

^c^ (STN1013001 – Latanoprost).

CI=confidence interval; N=number of observations; OAG/OHT=open-angle glaucoma/ocular hypertension.
