# Supplementary material for: Cost-Utility Analysis of STN1013001, a Latanoprost Cationic Emulsion, versus Other Latanoprost Formulations (Latanoprost) in Open-Angle Glaucoma or Ocular Hypertension and Ocular Surface Disease in France
Source: J Ophthalmol. 2022 Apr 29;2022:3837471. doi: 10.1155/2022/3837471 (PMC9076337; doi:10.1155/2022/3837471)
Supplement: Supplementary Materials — SText. Probabilistic sensitivity analysis: essential glossary Figure S1. Base case analysis-results-mean cost per patient per OAG/OHT stagea,b. Figure S2. Base case analysis-results-mean QALYs per patient per OAG/OHT stagea,b. Table S1. Base case analysis-methods-OAG/OHT staginga. Table S2. Base case analysis-methods-transition probability matrix (95% CI)a. Table S3. Base case analysis-results-OAG/OHT patients' age (range). Table S4. Base case analysis-results-mean number (SD) of OAG/OHT notional patients in each Markov state during a 5-year time horizon. Table S5. Base case analysis-results-adherence probabilities to OAG/OHT medications (95% CI)a,b. Table S6. Base case analysis-results-healthcare resource average consumption (95% CI)a-diagnosis. Table S7. Base case analysis-results-healthcare resource average consumption-management and follow-up-I-add-on therapies and drugs (range)a. Table S8. Base case analysis-results-healthcare resource average consumption (95% CI)a-management and follow-up-II-healthcare procedures and specialist visits. Table S9. Base case analysis-results-healthcare resource average consumption-OSD management-I-drugsa,b. Table S10. Base case analysis-results-healthcare resource average consumption (95% CI)a,b-OSD management-II-healthcare procedures and specialist visits. [file 3837471.f1.zip › Rev_3837471.f1/Rev_Supporting_Information_Table_S6_Journal_of_Ophthalmology(1).docx]

***Table S6*.** Base case analysis–results–healthcare resource average consumption (95% CI)^a^–diagnosis

| Cost items | STN1013001 | % targeted patients | Latanoprost | % targeted patients | Δ% targeted patients^b,c^ |
| --- | --- | --- | --- | --- | --- |
| OAG/OHT stages 0-5 | N=6185 |  | N=5700 |  |  |
| Healthcare procedures | | | | |  |
| Optical coherence tomography retinal nerve fiber layer | 0.71 (0.46; 1.01) | 70.51% | 0.68 (0.44; 0.98) | 68.49% | 2.02% (0.30%; 3.67%) |
| Gonioscopy | 1.18 (0.83; 1.59) | 64.74% | 1.19 (0.97; 1.43) | 65.75% | -1.01% (-2.72%; 0.70%) |
| Retinal nerve fibre thickness assessment | 0.47 (0.17; 0.93) | 29.49% | 0.51 (0.42; 0.60) | 31.51% | -2.02% (-3.66%; -0.36%) |
| Slit lamp examination | 1.18 (0.83; 1.59) | 100.00% | 1.19 (0.97; 1.43) | 100.00% | - |
| Tonometry | 1.18 (0.83; 1.59) | 100.00% | 1.19 (0.97; 1.43) | 100.00% | - |
| Visual field test | 1.18 (0.83; 1.59) | 82.37% | 1.19 (0.97; 1.43) | 82.88% | -0.50% (-1.85%; 0.86%) |
| Specialist visits | | | | |  |
| Ophthalmologist | 1.24 (1.02; 1.49) | 100.00% | 1.26 (1.05; 1.49) | 100.00% | - |

^a^ Unless otherwise specified, 95% CI was calculated assuming a Gamma probability distribution [14, 34].

^b^ (STN1013001 – Latanoprost).

^c^ 95% CI was calculated via the percentile method [34].

CI=confidence interval; N=number of observations; OAG/OHT=open-angle glaucoma/ocular hypertension.
