# Supplementary material for: Cost-Utility Analysis of STN1013001, a Latanoprost Cationic Emulsion, versus Other Latanoprost Formulations (Latanoprost) in Open-Angle Glaucoma or Ocular Hypertension and Ocular Surface Disease in France
Source: J Ophthalmol. 2022 Apr 29;2022:3837471. doi: 10.1155/2022/3837471 (PMC9076337; doi:10.1155/2022/3837471)
Supplement: Supplementary Materials — SText. Probabilistic sensitivity analysis: essential glossary Figure S1. Base case analysis-results-mean cost per patient per OAG/OHT stagea,b. Figure S2. Base case analysis-results-mean QALYs per patient per OAG/OHT stagea,b. Table S1. Base case analysis-methods-OAG/OHT staginga. Table S2. Base case analysis-methods-transition probability matrix (95% CI)a. Table S3. Base case analysis-results-OAG/OHT patients' age (range). Table S4. Base case analysis-results-mean number (SD) of OAG/OHT notional patients in each Markov state during a 5-year time horizon. Table S5. Base case analysis-results-adherence probabilities to OAG/OHT medications (95% CI)a,b. Table S6. Base case analysis-results-healthcare resource average consumption (95% CI)a-diagnosis. Table S7. Base case analysis-results-healthcare resource average consumption-management and follow-up-I-add-on therapies and drugs (range)a. Table S8. Base case analysis-results-healthcare resource average consumption (95% CI)a-management and follow-up-II-healthcare procedures and specialist visits. Table S9. Base case analysis-results-healthcare resource average consumption-OSD management-I-drugsa,b. Table S10. Base case analysis-results-healthcare resource average consumption (95% CI)a,b-OSD management-II-healthcare procedures and specialist visits. [file 3837471.f1.zip › Rev_3837471.f1/Rev_Supporting_Information_Table_S7_Journal_of_Ophthalmology(1).docx]

***Table S7*.** Base case analysis–results–healthcare resource average consumption–management and follow-up–I–add-on therapies–drugs (range)^a^

| Cost items | STN1013001 | Therapy duration^b^ | Latanoprost | Therapy duration^b^ |
| --- | --- | --- | --- | --- |
| OAG/OHT stage 0 | N=1560 |  | N=1460 |  |
| Probability of ≥1 add-on therapies | % targeted  patients: 40.00% |  | % targeted  patients: 40.00% |  |
| Brimonidine | 20.00% | 127.01 (0.00; 180.12) | 20.00% | 127.01 (0.00; 180.12) |
| Dorzolamide | 45.00% | 127.01 (0.00; 180.12) | 45.00% | 127.01 (0.00; 180.12) |
| Timolol | 35.00% | 234.71 (180.12; 365.25) | 35.00% | 238.45 (180.12; 365.25) |
| OAG/OHT stage 1 | N=1280 |  | N=1160 |  |
| Probability of ≥1 add-on therapies | % targeted  patients: 50.00% |  | % targeted  patients: 50.00% |  |
| Brimonidine | 15.00% | 98.50 (0.00; 365.25) | 15.00% | 93.17 (0.00; 180.12) |
| Brinzolamide | 10.00% | 22.83 (0.00; 365.25) | 10.00% | 25.19 (0.00; 365.25) |
| Dorzolamide | 20.00% | 184.11 (0.00; 365.25) | 20.00% | 187.63 (0.00; 365.25) |
| Timolol | 50.00% | 264.01 (180.12; 365.25) | 50.00% | 269.49 (180.12; 365.25) |
| Timolol+dorzolamide | 5.00% | 22.83 (0.00; 365.25) | 5.00% | 25.19 (0.00; 365.25) |
| OAG/OHT stage 2 | N=1280 |  | N=1550 |  |
| Probability of ≥1 add-on therapies | % targeted  patients: 60.00% |  | % targeted  patients: 60.00% |  |
| Brimonidine | 10.00% | 98.50 (0.00; 365.25) | 10.00% | 94.41 (0.00; 365.25) |
| Brinzolamide | 5.00% | 28.54 (0.00; 365.25) | 5.00% | 31.76 (0.00; 365.25) |
| Dorzolamide | 20.00% | 184.10 (0.00; 365.25) | 20.00% | 205.58 (0.00; 365.25) |
| Timolol | 60.00% | 264.01 (120.08; 180.12) | 60.00% | 237.34 (120.08; 180.12) |
| Timolol+dorzolamide | 5.00% | 28.54 (0.00; 365.25) | 5.00% | 31.76 (0.00; 365.25) |
| OAG/OHT stage 3 | N=1000 |  | N=930 |  |
| Probability of ≥1 add-on therapies | % targeted  patients: 70.00% |  | % targeted  patients: 70.00% |  |
| Brimonidine | 15.00% | 175.62 (0.00; 365.25) | 15.00% | 182.38 (0.00; 365.25) |
| Brinzolamide | 5.00% | 36.53 (0.00; 365.25) | 5.00% | 39.27 (0.00; 365.25) |
| Brinzolamide+brimonidine | 5.00% | 54.79 (0.00; 365.25) | 5.00% | 51.06 (0.00; 365.25) |
| Dorzolamide | 15.00% | 139.10 (0.00; 365.25) | 15.00% | 143.11 (0.00; 365.25) |
| Timolol | 50.00% | 175.62 (0.00; 365.25) | 50.00% | 182.38 (0.00; 365.25) |
| Timolol+dorzolamide | 10.00% | 91.31 (0.00; 365.25) | 10.00% | 90.33 (0.00; 365.25) |
| OAG/OHT stage 4 | N=650 |  | N=610 |  |
| Probability of ≥1 add-on therapies | % targeted  patients: 80.00% |  | % targeted  patients: 80.00% |  |
| Acetozolamide | 3.00% | 56.19 (0.00; 365.25) | 3.00% | 59.88 (0.00; 365.25) |
| Brimonidine | 5.00% | 210.99 (0.00; 365.25) | 5.00% | 204.89 (0.00; 365.25) |
| Brinzolamide | 2.00% | 56.19 (0.00; 365.25) | 2.00% | 59.88 (0.00; 365.25) |
| Brinzolamide+brimonidine | 5.00% | 73.05 (00.00; 365.25) | 5.00% | 59.88 (0.00; 365.25) |
| Dorzolamide | 15.00% | 137.94 (0.00; 365.25) | 15.00% | 145.02 (0.00; 365.25) |
| Timolol | 60.00% | 194.13 (0.00; 365.25) | 60.00% | 204.89 (0.00; 365.25) |
| Timolol+dorzolamide | 10.00% | 73.05 (00.00; 365.25) | 10.00% | 59.88 (00.00; 365.25) |
| OAG/OHT stage 5 | N=415 |  | N=390 |  |
| Probability of ≥1 add-on therapies | % targeted  patients: 95.00% |  | % targeted  patients: 95.00% |  |
| Acetozolamide | 5.00% | 302.26 (90.06; 365.25) | 5.00% | 301.74 (90.06; 365.25) |
| Brimonidine | 4.00% | 214.24 (0.00; 365.25) | 4.00% | 208.09 (0.00; 365.25) |
| Brinzolamide | 1.00% | 88.01 (0.00; 365.25) | 1.00% | 93.65 (0.00; 365.25) |
| Brinzolamide+brimonidine | 25.00% | 88.01(0.00; 365.25) | 25.00% | 74.92 (0.00; 365.25) |
| Dorzolamide | 1.00% | 126.23 (0.00; 365.25) | 1.00% | 133.17 (0.00; 365.25) |
| Timolol | 4.00% | 214.24 (0.00; 365.25) | 4.00% | 226.82 (0.00; 365.25) |
| Timolol+dorzolamide | 60.00% | 88.01 (0.00; 365.25) | 60.00% | 74.92 (0.00; 365.25) |

^a^one bottle per month assumed for all products; ^b^weighted average (days of therapy per year * proportions of patient receiving the therapy).

N=number of observations; OAG/OHT=open-angle glaucoma/ocular hypertension.
