# Supplementary material for: Cost-Utility Analysis of STN1013001, a Latanoprost Cationic Emulsion, versus Other Latanoprost Formulations (Latanoprost) in Open-Angle Glaucoma or Ocular Hypertension and Ocular Surface Disease in France
Source: J Ophthalmol. 2022 Apr 29;2022:3837471. doi: 10.1155/2022/3837471 (PMC9076337; doi:10.1155/2022/3837471)
Supplement: Supplementary Materials — SText. Probabilistic sensitivity analysis: essential glossary Figure S1. Base case analysis-results-mean cost per patient per OAG/OHT stagea,b. Figure S2. Base case analysis-results-mean QALYs per patient per OAG/OHT stagea,b. Table S1. Base case analysis-methods-OAG/OHT staginga. Table S2. Base case analysis-methods-transition probability matrix (95% CI)a. Table S3. Base case analysis-results-OAG/OHT patients' age (range). Table S4. Base case analysis-results-mean number (SD) of OAG/OHT notional patients in each Markov state during a 5-year time horizon. Table S5. Base case analysis-results-adherence probabilities to OAG/OHT medications (95% CI)a,b. Table S6. Base case analysis-results-healthcare resource average consumption (95% CI)a-diagnosis. Table S7. Base case analysis-results-healthcare resource average consumption-management and follow-up-I-add-on therapies and drugs (range)a. Table S8. Base case analysis-results-healthcare resource average consumption (95% CI)a-management and follow-up-II-healthcare procedures and specialist visits. Table S9. Base case analysis-results-healthcare resource average consumption-OSD management-I-drugsa,b. Table S10. Base case analysis-results-healthcare resource average consumption (95% CI)a,b-OSD management-II-healthcare procedures and specialist visits. [file 3837471.f1.zip › Rev_3837471.f1/Rev_Supporting_Information_Table_S8_Journal_of_Ophthalmology(1).docx]

***Table S8*.** Base case analysis–results–healthcare resource average consumption (95% CI)^a^–management and follow-up–II–healthcare procedures and specialist visits

| Cost items | STN1013001 | % targeted patients | Latanoprost | % targeted patients | Δ% targeted patients^b,c^ |
| --- | --- | --- | --- | --- | --- |
| OAG/OHT stage 0 | N=1560 |  | N=1460 |  |  |
| Healthcare procedures |  |  |  |  |  |
| Diurnal curve of intraocular pressure measurement | 0.71 (0.46; 1.01) | 7.05% | 0.68 (0.44; 0.98) | 6.85% | 0. 20% (-1.61%; 2.00%) |
| Gonioscopy | 0.29 (0.26; 0.34) | 21.79% | 0.32 (0.27; 0.36) | 23.29% | -1.49% (-4.45%; 1.42%) |
| Retinal nerve fibre thickness assessment | 1.96 (1.49; 2.50) | 100.00% | 1.96 (1.50; 2.47) | 100.00% | - |
| Slit lamp examination | 1.29 (1.08; 1.53) | 100.00% | 1.32 (1.11; 1.54) | 100.00% | - |
| Tonometry | 2.00 (1.53; 2.53) | 100.00% | 2.00 (1.55; 2.51) | 100.00% | - |
| Visual field test | 1.96 (1.49; 2.32) | 92.18% | 1.96 (1.50; 2.47) | 92.33% | -0. 15% (-2.08%; 1.76%) |
| Specialist visits |  |  |  |  |  |
| Ophthalmologist | 2.00 (1.53; 2.53) | 100.00% | 2.00 (1.55; 2.51) | 100.00% | - |
| OAG/OHT stage 1 | N=1280 |  | N=1160 |  |  |
| Healthcare procedures |  |  |  |  |  |
| Diurnal curve of intraocular pressure measurement | 0.45 (0.40; 0.51) | 10.94% | 0.48 (0.43; 0.54) | 10.34% | 0.59% (-1.85%; 3.03%) |
| Gonioscopy | 0.45 (0.40; 0.51) | 31.25% | 0.48 (0.43; 0.54) | 32.76% | -1.51% (-5.12%; 2.25%) |
| Retinal nerve fibre thickness assessment | 0.84 (0.73; 0.96) | 100.00% | 0.90 (0.78; 1.02) | 100.00% | - |
| Slit lamp examination | 0.91 (0.80; 1.02) | 100.00% | 0.97 (0.86; 1.08) | 100.00% | - |
| Tonometry | 0.91 (0.80; 1.02) | 100.00% | 0.97 (0.86; 1.08) | 100.00% | - |
| Visual field test | 0.84 (0.73; 0.96) | 94.53% | 0.90 (0.78; 1.02) | 94.83% | -0.30% (-2.12%; 1.48%) |
| Specialist visits |  |  |  |  |  |
| Ophthalmologist | 2.00 (1.67; 2.36) | 100.00% | 2.00 (1.69; 2.33) | 100.00% | - |
| OAG/OHT stage 2 | N=1280 |  | N=1150 |  |  |
| Healthcare procedures |  |  |  |  |  |
| Diurnal curve of intraocular pressure measurement | 0.55 (0.35; 0.78) | 10.94% | 0.52 (0.34; 0.75) | 10.43% | 0.50% (-2.04%; 2.90%) |
| Gonioscopy | 0.45 (0.41; 0.50) | 31.25% | 0.48 (0.43; 0.53) | 32.17% | -0.92% (-4.70%; 2.73%) |
| Retinal nerve fibre thickness assessment | 2.00 (1.67; 2.36) | 100.00% | 2.00 (1.70; 2.33) | 100.00% | - |
| Slit lamp examination | 1.45 (1.32; 1.59) | 100.00% | 1.48 (1.36; 1.60) | 100.00% | - |
| Tonometry | 2.00 (1.67; 2.36) | 100.00% | 2.00 (1.70; 2.33) | 100.00% | - |
| Visual field test | 2.00 (1.67; 2.36) | 94.53% | 2.00 (1.70; 2.33) | 94.78% | -0.25% (-2.07%; 1.54%) |
| Specialist visits |  |  |  |  |  |
| Ophthalmologist | 2.00 (1.67; 2.36) | 100.00% | 2.00 (1.70; 2.33) | 100.00% | - |
| OAG/OHT stage 3 | N=1000 |  | N=930 |  |  |
| Healthcare procedures |  |  |  |  |  |
| Diurnal curve of intraocular pressure measurement | 0.55 (0.36; 0.79) | 16.50% | 0.54 (0.35; 0.77) | 16.13% | 0.37% (-3.02%; 3.61%) |
| Gonioscopy | 0.45 (0.40; 0.50) | 33.00% | 0.46 (0.42; 0.51) | 33.33% | -0.33% (-4.57%; 3.78%) |
| Retinal nerve fibre thickness assessment | 2.00 (1.67; 2.36) | 89.00% | 2.00 (1.68; 2.35) | 89.25% | -0.25% (-2.99%; 2.50%) |
| Slit lamp examination | 1.45 (1.32; 1.59) | 100.00% | 1.46 (1.34; 1.59) | 100.00% | - |
| Tonometry | 2.00 (1.67; 2.36) | 100.00% | 2.00 (1.68; 2.35) | 100.00% | - |
| Visual field test | 2.00 (1.67; 2.36) | 100.00% | 2.00 (1.68; 2.35) | 100.00% | - |
| Specialist visits |  |  |  |  |  |
| Ophthalmologist | 2.10 (1.78; 2.45) | 100.00% | 2.11 (1.80; 2.44) | 100.00% | - |
| OAG/OHT stage 4 | N=650 |  | N=610 |  |  |
| Healthcare procedures |  |  |  |  |  |
| Diurnal curve of intraocular pressure measurement | 0.40 (0.26; 0.57) | 12.00% | 0.59 (0.53; 0.65) | 12.30% | -0.30% (-3.94%; 3.34%) |
| Gonioscopy | 0.60 (0.54; 0.67) | 45.23% | 0.41 (0.27; 0.59) | 43.28% | 1.95% (-3.64%; 7.50%) |
| Retinal nerve fibre thickness assessment | 2.35 (2.15; 2.57) | 84.00% | 2.33 (2.13; 2.53) | 83.61% | 0.39% (-3.70%; 4.48%) |
| Slit lamp examination | 1.95 (1.82; 2.09) | 100.00% | 1.92 (1.82; 2.02) | 100.00% | - |
| Tonometry | 2.60 (2.44; 2.76) | 100.00% | 2.46 (2.28; 2.64) | 100.00% | - |
| Visual field test | 2.35 (2.15; 2.57) | 100.00% | 2.33 (2.13; 2.53) | 100.00% | - |
| Specialist visits |  |  |  |  |  |
| Ophthalmologist | 3.00 (2.70; 3.31) | 100.00% | 3.00 (2.70; 3.32) | 100.00% | - |
| OAG/OHT stage 5 | N=415 |  | N=390 |  |  |
| Healthcare procedures |  |  |  |  |  |
| Diurnal curve of intraocular pressure measurement | 0.23 (0.15; 0.33) | 6.87% | 0.23 (0.15; 0.33) | 6.92% | -0.06% (-3.54%; 3.39%) |
| Gonioscopy | 0.77 (0.469; 0.86) | 59.76% | 0.77 (0.69; 0.86) | 58.46% | 1.30% (-5.51%; 8.11%) |
| Retinal nerve fibre thickness assessment | 2.48 (2.29; 2.68) | 86.27% | 2.46 (2.29; 2.64) | 86.15% | 0.11% (-3.37%; 3.27%) |
| Slit lamp examination | 2.25 (2.04; 2.48) | 100.00% | 2.23 (2.03; 2.44) | 100.00% | - |
| Tonometry | 2.77 (2.64; 2.91) | 100.00% | 2.62 (2.46; 2.77) | 100.00% | - |
| Visual field test | 2.77 (2.64; 2.91) | 100.00% | 2.62 (2.46; 2.77) | 100.00% | - |
| Specialist visits |  |  |  |  |  |
| Ophthalmologist | 3.00 (2.87; 3.14) | 20.00% | 3.00 (2.88; 3.12) | 20.00% | - |

^a^ Unless otherwise specified, 95% CI was calculated assuming a Gamma probability distribution [14, 34].

^b^ (STN1013001 – Latanoprost).

^c^ 95% CI was calculated via the percentile method [34].

CI=confidence interval; N=number of observations; OAG/OHT=open-angle glaucoma/ocular hypertension; OSD=ocular surface disease.
