# Supplementary material for: Cost-Utility Analysis of STN1013001, a Latanoprost Cationic Emulsion, versus Other Latanoprost Formulations (Latanoprost) in Open-Angle Glaucoma or Ocular Hypertension and Ocular Surface Disease in France
Source: J Ophthalmol. 2022 Apr 29;2022:3837471. doi: 10.1155/2022/3837471 (PMC9076337; doi:10.1155/2022/3837471)
Supplement: Supplementary Materials — SText. Probabilistic sensitivity analysis: essential glossary Figure S1. Base case analysis-results-mean cost per patient per OAG/OHT stagea,b. Figure S2. Base case analysis-results-mean QALYs per patient per OAG/OHT stagea,b. Table S1. Base case analysis-methods-OAG/OHT staginga. Table S2. Base case analysis-methods-transition probability matrix (95% CI)a. Table S3. Base case analysis-results-OAG/OHT patients' age (range). Table S4. Base case analysis-results-mean number (SD) of OAG/OHT notional patients in each Markov state during a 5-year time horizon. Table S5. Base case analysis-results-adherence probabilities to OAG/OHT medications (95% CI)a,b. Table S6. Base case analysis-results-healthcare resource average consumption (95% CI)a-diagnosis. Table S7. Base case analysis-results-healthcare resource average consumption-management and follow-up-I-add-on therapies and drugs (range)a. Table S8. Base case analysis-results-healthcare resource average consumption (95% CI)a-management and follow-up-II-healthcare procedures and specialist visits. Table S9. Base case analysis-results-healthcare resource average consumption-OSD management-I-drugsa,b. Table S10. Base case analysis-results-healthcare resource average consumption (95% CI)a,b-OSD management-II-healthcare procedures and specialist visits. [file 3837471.f1.zip › Rev_3837471.f1/Rev_Supporting_Information_Table_S9_Journal_of_Ophthalmology(1).docx]

***Table S9*.** Base case analysis–results–healthcare resource average consumption–OSD management– I–drugs^a,b^

| Cost items | STN1013001 | Latanoprost | Δ% targeted patients^c,d^ |
| --- | --- | --- | --- |
|  | % targeted  patients | % targeted  patients |  |
| OAG/OHT stage 0 | N=1560 | N=1460 |  |
| Preservative-free lubricant | 25.90% | 31.78% | -5.88% (-9.13%; -0.03%) |
| OAG/OHT stage 1 | N=1280 | N=1160 |  |
| Preservative-free lubricant | 35.94% | 47.93% | -11.99% (-15.82%;-0.08%) |
| OAG/OHT stage 2 | N=1280 | N=1550 |  |
| Preservative-free lubricant | 40.47% | 52.83% | -12.36% (-16.24%; -0.08%) |
| OAG/OHT stage 3 | N=1000 | N=930 |  |
| Preservative-free lubricant | 48.50% | 65.08% | - 9.47% (-14.77%; -0.04%) |
| OAG/OHT stage 4 | N=650 | N=610 |  |
| Preservative-free lubricant | 55.62% | 65.08% | - 9.47% (-14.77%; -0.04%) |
| Cyclosporin | 7.00% | 11.31% | -4.31% (-7.46%; -0.01%) |
| OAG/OHT stage 5 | N=415 | N=390 |  |
| Preservative-free lubricant | 60.96% | 60.75% | -11.34% (-17.68%; -0.05%) |
| Cyclosporin | 14.10% | 19.49% | -5.39% (-10.46%; -0.003%) |

^a^ One bottle per month assumed for all products.

^b^ Days of therapy per year =365.25 (range: 365.25;365.25).

^c^ (STN1013001 – Latanoprost); ^d^ 95% CI was calculated via the percentile method [34].

CI=confidence interval; N=number of observations; OAG/OHT=open-angle glaucoma/ocular hypertension; OSD=ocular surface disease.
